# Selective activation and expansion of regulatory T cells using lipid encapsulated mRNA encoding a long-acting IL-2 mutein

**DOI:** 10.1038/s41467-022-31130-9

**Published:** 2022-07-05

**Authors:** Seymour de Picciotto, Nicholas DeVita, Chiaowen Joyce Hsiao, Christopher Honan, Sze-Wah Tse, Mychael Nguyen, Joseph D. Ferrari, Wei Zheng, Brian T. Wipke, Eric Huang

**Affiliations:** Moderna, Inc, Cambridge, MA 02139 USA

**Keywords:** Regulatory T cells, Interleukins, Autoimmunity, Systems analysis

## Abstract

Interleukin-2 (IL-2) is critical for regulatory T cell (Treg) function and homeostasis. At low doses, IL-2 can suppress immune pathologies by expanding Tregs that constitutively express the high affinity IL-2Rα subunit. However, even low dose IL-2, signaling through the IL2-Rβ/γ complex, may lead to the activation of proinflammatory, non-Treg T cells, so improving specificity toward Tregs may be desirable. Here we use messenger RNAs (mRNA) to encode a half-life-extended human IL-2 mutein (HSA-IL2m) with mutations promoting reliance on IL-2Rα. Our data show that IL-2 mutein subcutaneous delivery as lipid-encapsulated mRNA nanoparticles selectively activates and expands Tregs in mice and non-human primates, and also reduces disease severity in mouse models of acute graft versus host disease and experimental autoimmune encephalomyelitis. Single cell RNA-sequencing of mouse splenic CD4^+^ T cells identifies multiple Treg states with distinct response dynamics following IL-2 mutein treatment. Our results thus demonstrate the potential of mRNA-encoded HSA-IL2m immunotherapy to treat autoimmune diseases.

## Abstract (web summary)

IL-2 has been used to expand regulatory T (Treg) cells for treating inflammatory disorders. Here the authors test an engineered IL-2 mutein, delivered subcutaneously as mRNA, to show its increased specificity for activating and expanding Treg cells in both rodents and non-human primates, and to demonstrate its ability to suppress autoimmunity in mouse models.

## Introduction

Interleukin-2 is critical for T cell growth, survival, and function, and thus maintaining immune homeostasis. First discovered in 1983^1^, it was rapidly translated to the clinic in a high dose treatment regimen for renal cell carcinoma and melanoma in 1984^2^. While it gained approval in 1992 and became the first immunotherapy, later studies demonstrated its essential role in the survival and proliferation of Tregs^3,4^. Thus, it became apparent that IL-2 preferentially stimulated Tregs at low concentrations, but it could stimulate all T and natural killer (NK) lymphocytes at higher concentrations. This dichotomy stems from two forms of the IL-2 receptor with differing affinities: IL-2 can bind the IL-2 receptor beta (IL2RB, CD122) and the gamma common chain (γc, CD132) with an equilibrium dissociation constant (*K*_D_) ~1 nM. This intermediate affinity receptor is expressed by many cells of the immune system. However, regulatory T cells constitutively express the IL-2 receptor alpha chain (IL2RA, CD25) which allows the formation of the heterotrimeric receptor with a higher affinity (*K*_D_ ~ 10 pM), making them more sensitive to IL-2. Signaling is mediated by IL2RB and γc, initiated by the phosphorylation of JAK1/3 and resulting in the activation of STAT5, MAPK and AKT pathways. As such, IL-2 can induce the proliferation of T and NK cells and promote the differentiation of Th1 and Th2 CD4+ T Cells.

Regulatory T cells represent a subset of CD4+ T cells characterized by the expression of the lineage determining transcription factor FOXP3. They possess contact-dependent and independent mechanisms that make them crucial to the maintenance of peripheral tolerance and dampening autoimmune responses. Early studies have shown that Treg are defective in number and/or function in various human autoimmune conditions such as systemic lupus erythematosus^5^, rheumatoid arthritis^6^, type I diabetes^7,8^ and others^9,10^. These results suggest that interventions that increase the number of functional Tregs in patients can improve disease outcome^11,12^. Since low doses of IL-2 can selectively expand Tregs, clinical trials have been conducted in chronic graft vs host disease^13^ (cGvHD), systemic lupus erythematosus^14^ and several other autoimmune conditions^15–18^. A study with steroid-refractory cGvHD patients receiving daily subcutaneous (SC) low dose IL-2 reported that 20 of 33 had partial responses^19^, which was encouraging given the poor prognosis of this disease^20^. While most of these studies were not placebo controlled, the observed Treg expansion and reduction in disease scores nonetheless generated broad enthusiasm in the field. However, current low-dose IL-2 therapy has some limitations, including dose-limiting pro-inflammatory effects on various cell populations and poor pharmacokinetics^21^. To address these, several protein engineering strategies have been proposed to increase half-life and Treg selectivity such as complexation with antibody^22,23^, mutations^24,25^ and de novo cytokine design^26^. Generally, these approaches weaken the interactions of IL-2 protein with IL2RB either through destabilizing mutations or via epitope masking^27^, thus increasing reliance upon IL2RA interactions.

mRNA delivery is emerging as a promising technology for many diseases, with several trials ongoing and the expression of a variety of proteins has been demonstrated preclinically^28,29^ and clinically^30,31^. In this study, we hypothesized that an extended half-life IL-2 mutein engineered for enhanced selectivity of the trimeric over the dimeric IL-2 receptor could selectively expand Tregs in vivo when delivered by a lipid encapsulated messenger RNA (mRNA).

We introduce three mutations in human IL-2 as a fusion with serum albumin (HSA-IL2m). In vitro, we observe STAT5 phosphorylation exclusively in Tregs, differentiating it from wildtype IL-2 fusion (HSA-IL2wt) that induces STAT5 phosphorylation in Tregs, NK and conventional T cells (Tcon). In mice, rats and non-human primates, *HSA-IL2m* (italicized to indicate mRNA instead of protein) selectively expands Tregs without the activation of NK and conventional T cells observed with *HSA-IL2wt*. Finally, *HSA-IL2m* provides specific sustained activation and expansion of Tregs, resulting in reduced disease severity in mouse models of graft vs host disease and experimental autoimmune encephalomyelitis (EAE). Based on these results, we propose that this strategy has the potential to dampen autoreactive T cells and restore immune balance in several autoimmune disorders.

## Results

### HSA-IL2m leads to STAT5 phosphorylation exclusively in Tregs

**Fig. 1:**
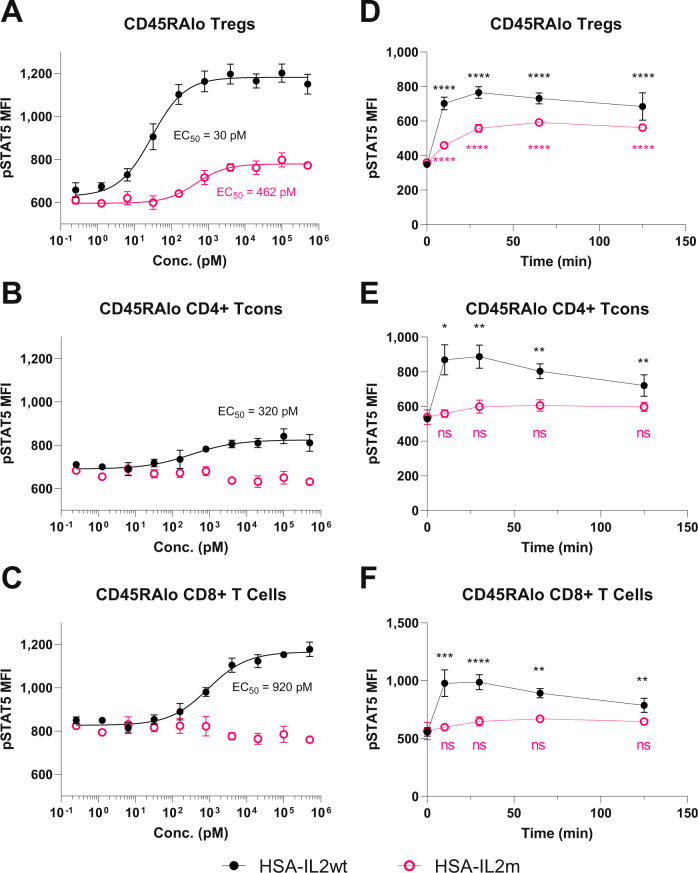
Comparison of STAT5 phosphorylation in various human T cells subsets after treatment with HSA-L2wt and HSA-IL2m. HSA-IL2wt and HSA-IL2m titrations on **A** memory Tregs, **B** memory CD4+ conventional T cells, and **C** memory CD8+ T cells after 30 min incubation. Kinetics of STAT5 phosphorylation with 10 nM of HSA-IL2wt and HSA-IL2m on **D** memory Tregs, **E** memory CD4+ conventional T cells, and **F** memory CD8+ T cells. Data are presented as mean ± s.d. EC50 are derived from log-logistic fit. Statistical analysis from two-way ANOVA with Dunnet’s multiple comparison to baseline, two-sided (*p*-value * < 0.05, ** < 0.01, *** < 0.001, **** < 1e−4, ns = not significant, *n* = 4). Source data are provided as a Source Data file.

To assess the functionality of HSA-IL2m protein, a comparison was made on its ability to induce STAT5 phosphorylation in human PBMC relative to HSA-IL2wt. Given that Tregs constitutively express the high affinity receptor CD25 (IL2RA), STAT5 phosphorylation was measured in Tregs as well as in conventional CD4 and CD8 T cells that express the intermediate affinity IL2R complex. After a 30-minute incubation, all T cells responded (STAT5 phosphorylation) to stimulation with HSA-IL2wt whereas only Tregs responded to HSA-IL2m. The EC50 on Treg was 30 and 460 pM for HSA-IL2wt and HSA-IL2m, respectively (Fig. 1A). However, EC50 of HSA-IL2wt was 320 and 920 pM on CD4 and CD8+ T cells (Fig. 1B, C). Stimulation of cynomolgus PBMC, as well as mouse and rat splenocytes resulted in similar observations (Supplementary Fig. 1). Thus, in all evaluated species, HSA-IL2m stimulated phosphorylation of STAT5 only in Tregs.

To extend these observations, human PBMC were exposed to 10 nM of HSA-IL2wt and HSA-IL2m and the kinetics of STAT5 phosphorylation were measured (Fig. 1D–F). HSA-IL2wt stimulation led to rapid STAT5 phosphorylation in all T cell subsets examined, reaching a maximum after 10 min and slowly declining after 30 min. In contrast, HSA-IL2m induced STAT5 phosphorylation only in Tregs. Moreover, peak phosphorylation was delayed to 60 min. Taken together, these data demonstrate the selectivity of HSA-IL2m for Tregs in vitro.

### Sustained in vivo Treg expansion and activation following subcutaneous delivery of LNP encapsulated mRNA encoding HSA-IL2m

**Fig. 2:**
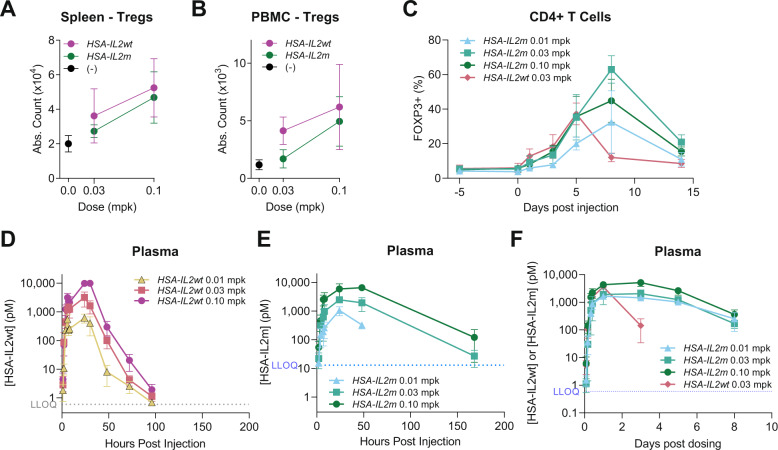
Extended Treg expansion with *HSA-IL2m* treatment. Treg counts in **A** spleen and **B** blood of C57BL/6 female mice following treatment with *HSA-IL2wt* and *HSA-IL2m* (*n* = 5). **C** Time course of Treg frequency among CD4+ T cells in blood of cynomolgus monkey following treatment with *HSA-IL2m* and *HSA-IL2wt* (*n* = 4). Plasma concentration of **D** HSA-IL2wt and **E** HSA-IL2m in C57BL/6 mice (*n* = 5). **F** Plasma concentration of HSA-IL2wt and HSA-IL2m in cynomolgus monkey (*n* = 4). Data are presented as mean ± s.d. LLOQ = Lowest limit of quantification. Source data are provided as a Source Data file.

Formulated mRNA encoding HSA-IL2m and HSA-IL2wt were assessed for their ability to expand Tregs in vivo. Lipid nanoparticle encapsulated *HSA-IL2wt* or *HSA-IL2m* were subcutaneously administered into C57BL/6 mice and cynomolgus monkeys. In mice, *HSA-IL2wt* increased the absolute number of Treg in blood and spleen. The response was dose-dependent and maximal 4 days post administration (Supplementary Fig. 2A, B). At dose levels of 0.03 and 0.1 mg per kg (mpk), the Treg expansion was comparable in spleen for *HSA-IL2wt* and *HSA-IL2m* (Fig. 2A). In blood, Tregs were expanded with 0.03 and 0.1mpk of *HSA-IL2wt* but required 0.1 mpk of *HSA-IL2m* (Fig. 2B). In cynomolgus monkeys, Tregs comprised up to 40% of circulating CD4+ T cells at 5 days post injection with *HSA-IL2wt*, but sharply declined thereafter (Fig. 2C). In contrast, treatment with *HSA-IL2m* prolonged Treg expansion, and reached a maximum at 8 days (Fig. 2C). Moreover, the Treg frequency within the CD4+ T cell compartment in blood was 2–4-fold higher with *HSA-IL2m* two weeks after treatment, whereas the difference from baseline was not statistically significant for *HSA-IL2wt* (Supplementary Fig. 3A). This expansion/contraction was reproducible over a period of three rounds of biweekly dosing (Supplementary Fig. 3B).

This study hypothesized that the extended duration of Treg expansion was due to prolonged plasma half-life of HSA-IL2m protein compared to HSA-IL2wt as a result of reduced consumption by non-Treg cells. In mice, plasma levels of HSA-IL2m were still detectable one-week post injection (Fig. 2E), while HSA-IL2wt levels were below the detection limit 4 days post injection (Fig. 2D). Similarly, in cynomolgus monkey, the HSA-IL2wt plasma levels declined 25-fold between 24 and 72 h post dosing, whereas HSA-IL2m was reduced 11-fold for between 24 h and 8 days post dosing (Fig. 2F). Taken together, these results indicate that *HSA-IL2m* mRNA treatment results in prolonged Treg expansion in rodent and non-human primates and correlates with longer half-life of the mutein, possibly due to reduced consumption.

### Tregs from *HSA-IL2m* treated animals have enhanced suppressive capacity

**Fig. 3:**
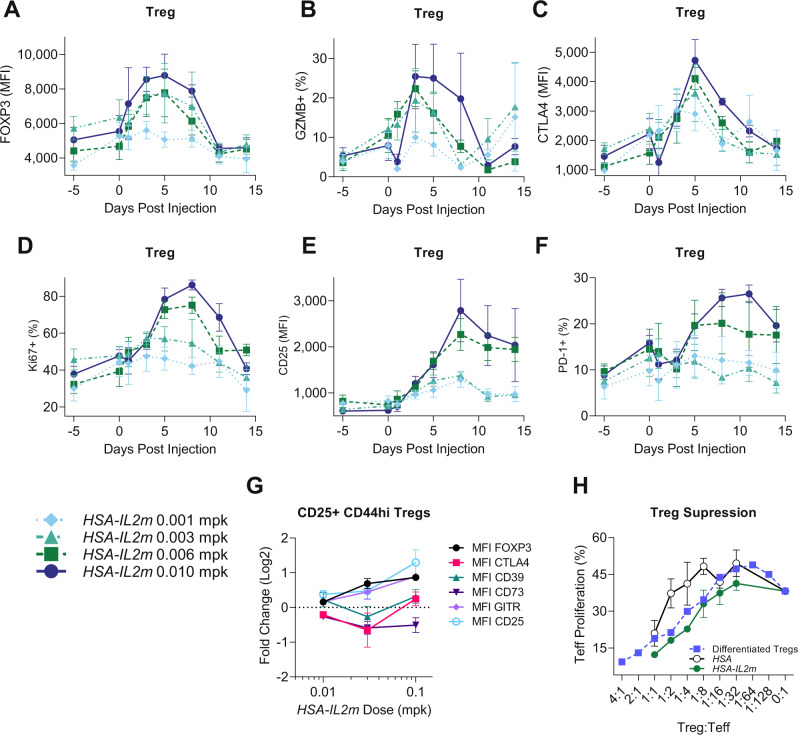
Characterization of regulatory T cells in mouse and cynomolgus monkey following *HSA-IL2m* treatment. **A** FOXP3 MFI, **B** frequency of GZMB+, **C** MFI of CTLA-4, **D** Frequency of Ki67+, **E** CD25 MFI, **F** frequency of PD-1+ in cynomolgus monkey Tregs following *HSA-IL2m* treatment (*n* = 4). **G** median fluorescence intensity of FOXP3, CTLA4, CD39, CD73 and GITR in murine Treg following *HSA-IL2m* treatment in C57BL/6 mice (*n* = 6). **H** Suppression of conventional T cells by regulatory T cells from control mice (*HSA*), *HSA-IL2m*, and in vitro differentiated Tregs (*n* = 3). Data are presented as mean ± s.e. (**A**–**F**) and mean ± s.d. **G**–**H** Source data are provided as a Source Data file.

Having demonstrated that Tregs were expanded with *HSA-IL2m* treatment, the next step was to investigate their phenotype and functionality. Cynomolgus monkey were monitored for the expression of several Treg activation (CD25, FOXP3), proliferation (Ki67), and effector (GZMB, PD-1) markers by flow cytometry after treatment with *HSA-IL2m*. These markers were upregulated in a dose-dependent fashion but with distinct kinetics (Fig. 3A–F). FOXP3 (Fig. 3A), GZMB (Fig. 3B) and CTLA4 (Fig. 3C) followed a similar early upregulation pattern with maximum signal 5 days post injection and returned to baseline after 2 weeks. Ki67 also upregulated rapidly but had an extended peak at 8 days (Fig. 3D). CD25 (Fig. 3E) and PD-1 (Fig. 3F) expression were upregulated and remained elevated two weeks after dosing. These results suggest a decoupling between the IL-2 signaling threshold required for proliferation and activation.

We also characterized Tregs from *HSA-IL2m* treated mice 2 days after treatment and observed upregulation of activation and effector molecules CD25, FOXP3, CD39, CD73, GITR, and CTLA4 (Fig. 3G). To confirm whether this phenotype translated in enhanced functionality, we tested the ability of Tregs to suppress conventional T cells in the presence of antigen-presenting cells and anti-CD3 antibody. Tregs from *HSA-IL2m* treated animals had a greater suppressive ability than Tregs from *HSA* treated control animals (Fig. 3H). Thus, *HSA-IL2m* treatment leads to the expansion and activation of Tregs, with correspondingly greater ability to inhibit T cell responses.

### *HSA-IL2m* is selective to Treg activation in mice and non-human primates

**Fig. 4:**
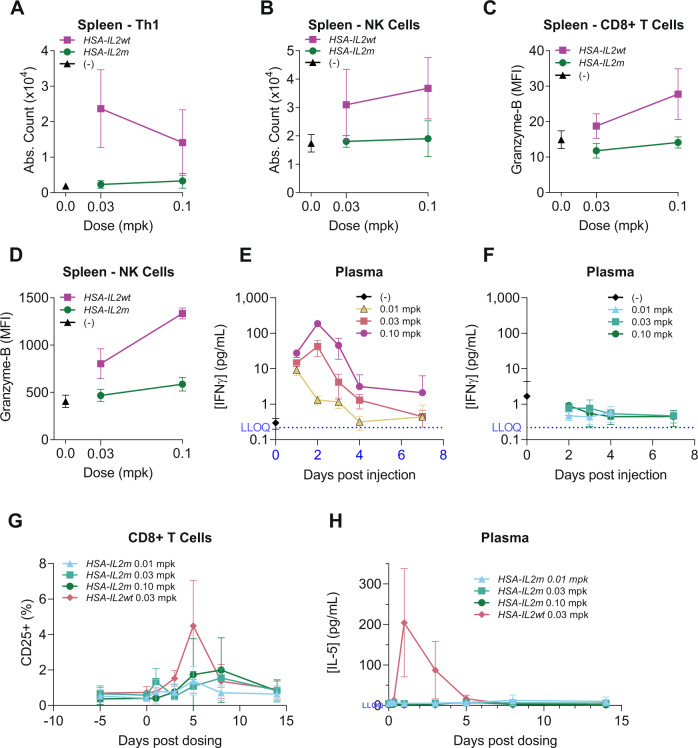
In vivo enhanced selectivity of *HSA-IL2m* towards regulatory T cells in mice and non-human primates. In mice, *HSA-IL2wt* expands proinflammatory **A** Th1 cells (defined as T cells expressing T-bet+ and CD4+) and **B** NK cells in spleen and activates **C** CD8 T cells and **D** NK cells as shown by upregulation of GZMB (*n* = 5). Plasma concentration of IFNγ after administration of **E** *HSA-IL2wt* or **F** *HSA-IL2m* (*n* = 5). In cynomolgus monkey, *HSA-IL2wt* activates CD8+ T cells (**G**) and stimulates the production of IL-5 (**H**) whereas *HSA-IL2m* does not at the equivalent and higher doses (*n* = 4). Data are presented as mean ± s.d. Source data are provided as a Source Data file.

A major drawback of IL-2 therapy in the context of autoimmune diseases is the activation of proinflammatory cells. We assessed the effects of *HSA-IL2m* on other IL-2 responsive immune cells, contrasting it with *HSA-IL2wt*. In mice, while *HSA-IL2m* and *HSA-IL2wt* treatment induced comparable proliferation of Tregs in blood and spleen (Fig. 2A, B), the number of Th1 (Fig. 4A and Supplementary Fig. 4A) and NK cells (Fig. 4B and Supplementary Fig. 4B) were greatly increased with *HSA-IL2wt* treatment but not with *HSA-IL2m*. Consistently, expression of proliferation marker Ki67 was elevated in Tregs but not in Tcons with *HSA-IL2m* (Supplementary Fig. 4C–F). Furthermore, CD8+ T cells and NK cells had increased GZMB expression only in the *HSA-IL2wt* treated mice (Fig. 4C, D). The same observations were found in peripheral blood cells (Supplementary Fig. 4G, H). In plasma, pro-inflammatory cytokines IFNγ (Fig. 4E, F), IL-5 (Supplementary Fig. 4I, J) and IL-9 (Supplementary Fig. 4K, L) were elevated with *HSA-IL2wt* treatment, whereas these cytokines remained at baseline with *HSA-IL2m* treatment. In cynomolgus monkey, while 0.03 mpk of *HSA-IL2wt* and *HSA-IL2m* led to a similar initial Treg expansion, *HSA-IL2wt* treatment also upregulated CD25 on CD8+ T cells (Fig. 4G). However, even at the 3.3-fold higher dose of 0.1 mpk of *HSA-IL2m*, we did not observe activation of CD8 T cells (determined by lack of CD25 upregulation, Fig. 4G). In plasma, IL-5 concentration was elevated with *HSA-IL2wt* treatment but not with *HSA-IL2m* (Fig. 4H). Taken together, these results suggest the effects of *HSA-IL2m* are specific to Tregs in mice and cynomolgus monkey under the conditions tested.

### Single cell RNA sequencing identifies Treg subsets with distinct IL-2 signaling requirements

**Fig. 5:**
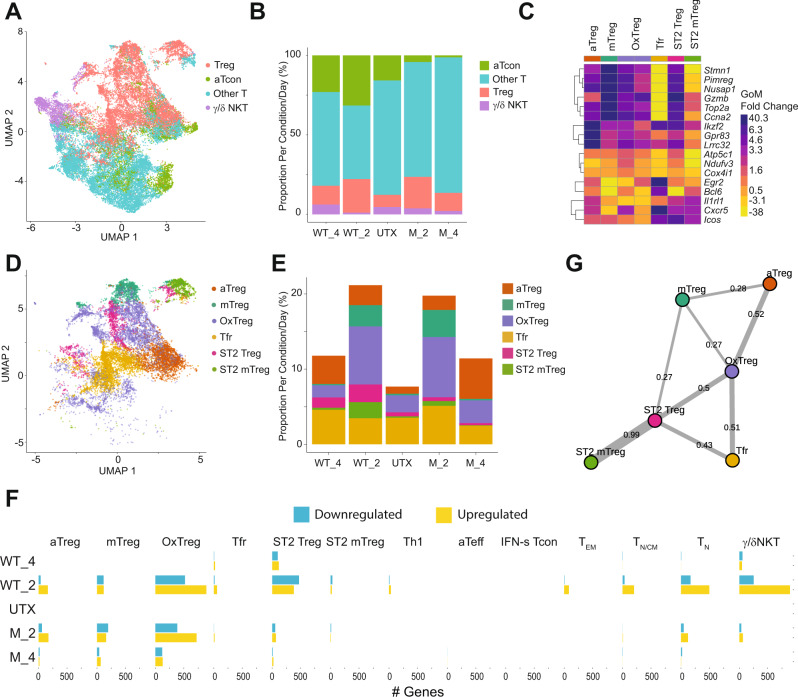
Single cell transcriptomic of splenic CD4 T cells in naïve and HSA-IL2wt/m treated mice. Cells were harvested either 2- or 4-days post-treatment from *n* = 2 mice per treatment condition. **A** Uniform manifold approximation and projection (UMAP) embedding of single-cell gene expression profiles (dots) colored by family. **B** Frequency of cells by family assignment and treatment condition (UTX: Untreated; WT_2, WT_4: Day 2 and Day 4 after *HSA-IL2wt*; M_2, M_4: Day 2 and Day 4) after *HSA-IL2m* treatment. **C** Differentially-expressed genes characterizing each Treg topic. Color corresponds to log2-fold-change of mean gene expression for all cells assigned to topic k vs all other topics weighted by membership weights. **D** UMAP embedding of single-cell expression profile colored by Treg topics. **E** Frequency of Treg topics colored by treatment condition. **F** Treatment induced differentially-expressed genes by topics. **G** Treg cell state association graph reconstructed using partition-based graph abstraction (PAGA). Edge width corresponds to connectivity between Treg topics. Source data are provided as a Source Data file.

While the terminology of Tregs generally refers to FOXP3+ CD25+ CD4+ T cells, an increasing number of reports suggest a variety of Treg subsets^32,33^. Therefore, we determined whether IL-2 expands and uniformly activates these various subsets. To further characterize and differentiate the effects of *HSA-IL2m* to *HSA-IL2wt*, we undertook a single cell transcriptomic analysis of splenic CD4+ T cells from *HSA-IL2m* and *HSA-IL2wt* treated mice, and untreated mice for baseline comparison. The enriched CD4+ T cells were 98.3 % pure based on flow cytometry evaluation. The dataset contained 164,240 cells with uniform representation across samples. Topic modeling^34^ with *K* = 18 was performed and expression of canonical CD4+ T cells gene were evaluated (Supplementary Fig. 5A). Two topics had a much lower expression of *Cd3e*, *Cd3d,* and *Cd4*. They represented a total of 981 cells (0.6%) and were enriched for genes typically associated with B cells and myeloid cells. These two topics were excluded from further analysis, attributing them to cells that escaped the purification method. Based on differential gene expression profile we grouped the remaining topics into 4 families: Treg, activated Tcon, other Tcon, and γ/δ NKT cells (Fig. 5A and Supplementary Fig. 5B).

Both *HSA-IL2wt* and *HSA-IL2m* treated samples showed a similar increase in Treg abundance, but only *HSA-IL2wt* increased the abundance of the activated Tcon family (Fig. 5B and Supplementary Fig. 6). The Treg family consisted of 6 topics all sharing expression of genes commonly associated with Tregs (Supplementary Fig. 7A) yet had distinct differentially expressed genes (Fig. 5C) and changes in abundance with treatment (Fig. 5E and Supplementary Fig. 8). Topic 18, representing follicular Tregs (Tfr), did not change substantially in abundance with treatment (Fig. 5E). It was characterized by higher expression of *Cxcr5*, *Bcl6*, *Icos*, and *Egr2* (Fig. 5C)*.* This population had the lowest *Il2ra* expression levels of all Treg topics (Supplementary Fig. 7A) and it was the most abundant population in untreated animals. Topic 16/17 was defined by upregulation of transcripts involved in oxidative phosphorylation (*Atp5c1*, *Atp5h*, *Atp5l*, *Atp5g1*, *Cox4i1*, *Cox6c*, *Ndufv3*, *Ndufb5*, and *Ndufs2*) and actin regulation (*Tmsb4x*, *Tmsb10*, *Cotl1*, *Abracl*, *Pfn1*, and *Capzb)* thus receiving the label of OxPhos Treg (OxTreg). Topic 5 was defined by high expression of common Treg genes (*Gpr83*, *Ikzf2*, *Foxp3*, *Lrrc32*, *Ikzf4*) and *Bcl2* suggesting an activated Treg state (aTreg). Topic 6 was characterized by transcripts associated with cytoskeletal reorganization (*Nusap1*, *Tpx2*, *Aurkb*, *Tubb5*, *Stmn1*, *Tuba1b*, and *Tpx2*), mitosis (*Ccna2*, *Pclaf*, *Spc25*, *Plk1*, *Rrm2*, *Pimreg*, *Top2a*) and proliferation (*Mki67*, *Top2a*), thus defining it as a mitotic Treg (mTreg). The OxTreg, aTreg, and mTreg were expanded by both *HSA-IL2m* and *HSA-IL2wt* treatments.

The OxTreg and mTreg populations were rapidly expanded by day 2 and rapidly declined by day 4 after treatment. In contrast, the aTreg population had a slower but more sustained expansion (Fig. 5E). Topic 1 and 15 were expanded with *HSA-IL2wt* treatment only. They were characterized by the expression of several transcripts associated with Treg activation and effector functions (*Ass1*, *Klrg1*, *Gzmb*, *Tagln2*, *Tnfrfs9*, *Lgals1*, *icos*, and *s100a6*). Furthermore, they expressed *il1rl1* and *il-13*, thus defining these populations as ST2+ Tregs (ST2 Treg), a previously reported highly suppressive Treg subset^35^. Topic 1 was distinct from 15 by the expression of genes associated with cell cycle regulation, thus clarifying its label as ST2 mitotic Treg (mTreg). Given the role of IL-2 signaling in Treg proliferation, we applied the supervised method Cyclone^36^ to assign each single cell to a discrete cell cycle stage: G1, S, G2.M. A large fraction of the mTreg and ST2 mTreg were assigned to G2M (Supplementary Fig. 7B), confirming their mitotic state. Taken together, the data on Tregs identified 3 different states (mitotic, oxidative phosphorylation, activated) and 3 subsets (ST2, Tfr and regular). Tfr were insensitive to treatments, ST2 were preferentially expanded by *HSA-IL2wt* only, while the other cell type/states responded equally to both treatments. In both treatments, the mitotic and OxPhos states were rapidly expanded or induced while the activated state was slower and more sustained. To explore the connectivity between these Treg subsets, we performed Partition-based graph abstraction on the Treg cells. The associations suggest that the various types and states can arise from OxTreg (Fig. 5G) and the decline in mTregs and OxTregs could be associated with the expansion of the aTregs.

To validate these findings, we selected markers that were differentially expressed in these Treg subsets for flow cytometry characterization. For Tfr, we used the surface chemokine receptor CXCR5 and the transcription factor BCL6. For ST2 Tregs, we chose ST2, KLRG1 and ITGB1. For aTreg we used CD25hi and GARP. The OxPhos Treg did not contain differentially expressed genes for which we could find commercially available flow cytometry antibodies. Using flow cytometry, we could identify distinct populations reflected by these markers (Supplementary Fig. 9A). At 0.05 mpk, the total Treg expansion was comparable between both mRNA treatments (Supplementary Fig. 9B). Tfr were indeed insensitive to treatments (Supplementary Fig. 9C). Furthermore, the sustained expansion of aTregs was also recapitulated (Supplementary Fig. 9D). At the same dose level, while total Treg (Supplementary Fig. 9B) and aTreg (Supplementary Fig. 9D) expansion was comparable, we confirmed that ST2 Tregs (Supplementary Fig. 9E) were more strongly expanded by *HSA-IL2wt*. However, at the higher dose of 0.2 mpk, *HSA-IL2m* expansion of ST2 Tregs was comparable to *HSA-IL2wt*. To investigate the mitotic states, we used the proliferation marker Ki67 (Supplementary Fig. 9E–G) and a thymidine analog, 5-ethynyl-2′-deoxyuridine (EdU), incorporation assay. EdU incorporation in Tregs was greatest 2 days post injection and returned close to baseline by 4 days post injection (Supplementary Fig. 10), confirming the single cell RNA sequencing findings. These results from single cell RNA sequencing and flow cytometry indicate that subsets of Tregs have distinct dynamics and qualitative responses to IL-2 stimulation.

### Single cell RNA sequencing highlights specificity of mutein toward Tregs

*HSA-IL2wt* increased the abundance of the activated Tcon family (Fig. 5B and Supplementary Fig. 6), while they were underrepresented in *HSA-IL2m* treated mice. This family consisted of three topics that were enriched in interferon gamma and alpha response pathway (Supplementary Fig. 5C). Topic 3 was characterized by ribosomal transcripts (*Uba52, Rps27, Rpl9*, *Rpl13a*, *Rps12*, *Eerf1a1*, and *Eef2*), defining them as activated effector T cells (aTeff). Topic 11 was characterized by a large set of interferon stimulated genes (*Ifit3, Ifi27l2a*, Ifit3b, *Stat1*, *Ifit1*, and *Isg15*), thus, referred to as IFN-stimulated CD4+ (IFN-s Tcon). Topic 13 was also characterized by high expression of ribosomal transcripts (*Rps6, Rpl13,* and *Rpl3*) and *Klrb1*. Given its abundance and previous flow cytometry data, we identified this topic as type 1 helper T cells (Th1). Treatment with *HSA-IL2wt* markedly increased the fraction of the Th1 and IFN-s topics (Supplementary Fig. 11). In contrast, the aTeff topic did not change in abundance with *HSA-IL2wt* but was strikingly reduced in the *HSA-IL2m* conditions.

Topic 4 was characterized by expression of *Tbx21*, *Ifng*, *Il4*, and *Cxcr6* (Supplementary Fig. 5B). In addition, this topic had differential expression of TCR receptor gamma/delta components (*Trdc*, *Tcrg-C2*, and *Tcrg-C4)*, killer cell lectin like receptor (*Klra1*, *Klra3*, *Klrb1*, *Klrc1*, and *Klrd1), and Zbtb16* which encodes for the transcription factor PLZF. This transcriptional signature is suggestive of a gamma/delta NKT cell subset^37^. The remaining T cell topics represented naïve (T_N_), effector memory (TEM) and a heterogenous mix of naïve and central memory T cells (T_N/CM_) which did not meaningfully change in abundance with treatment and were grouped in the “Other T” family (Supplementary Fig. 12).

This differential abundance analysis is reflective of the proliferative and survival responses of these individual cell types and cell states from IL-2 treatments. To further characterize this responsiveness, we carried out a differential state analysis, focusing on transcriptional changes of a given cell state with treatment. We measured the number of genes that were significantly up or down regulated (FDR < 0.05) at 2- and 4-days post treatment with *HSA-IL2m* or *HSA-IL2wt* compared to baseline (Fig. 5F). Transcriptional state was modulated in Tregs, conventional T cells and γ/δNKT with *HSA-IL2wt* treatment, while transcriptional changes induced by *HSA-IL2m* were more focused on Treg. Three Treg subsets (aTreg, mTreg and OxTreg) had similar differentially expressed genes two days after *HSA-IL2m* and *HSA-IL2wt* treatment; notably the signaling effects were prolonged for *HSA-IL2m* (Fig. 5F), presumably due to the longer exposure of the HSA-IL2m protein. The ST2 Treg topic was more responsive to *HSA-IL2wt* treatment and the Tfr was relatively insensitive to treatments. These results were aligned with the differential abundance analysis. In conclusion, *HSA-IL2wt* treatment signaled through regulatory and conventional T cells and resulted in increased abundance of topics associated with IFN response. In contrast, the effects of *HSA-IL2m* were restricted to Tregs.

### *HSA-IL2m* reduces disease severity in murine acute graft versus host disease and experimental autoimmune encephalitis

**Fig. 6:**
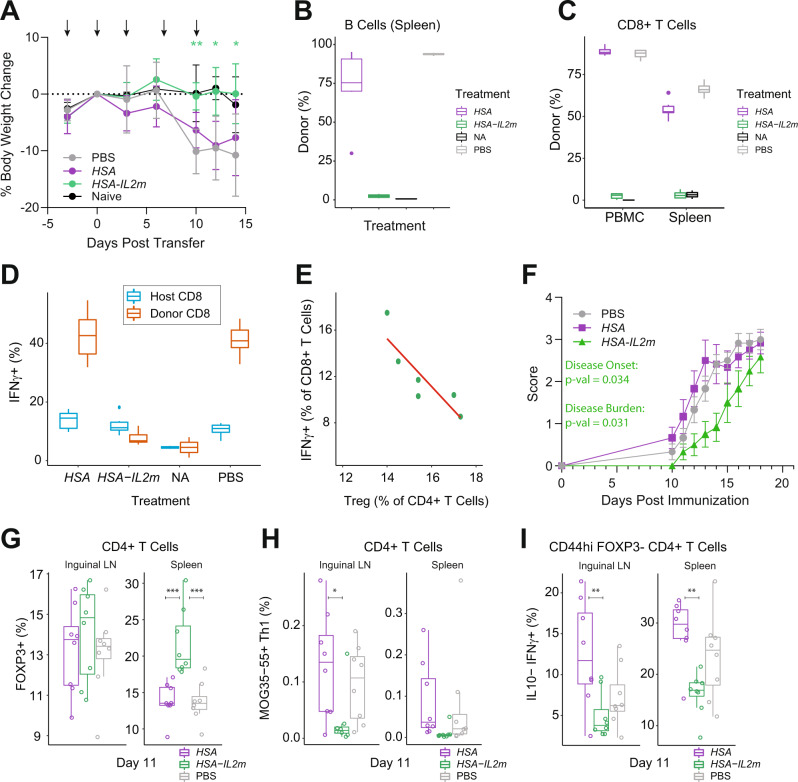
IL2 mutein is efficacious in acute GvHD and EAE mouse models. Acute GvHD model: **A** Body weights of F1 animals following adoptive transfer of B6 cells. Animals (*n* = 6 for all groups except *n* = 2 for NA group) were dosed on days −3, 0, 3, 7 and 10 as indicated by arrows. Asterisks denote significance (two-way ANOVA, two-sided, *p*-value * < 0.05, ** < 0.01) compared to PBS group and data are presented as mean ± s.d. Frequency of donor **B** B cells and **C** CD8 T cells. **D** Frequency of IFNγ producing CD8 T cells. **E** correlation of Treg frequency and CD8 T cell IFNγ expression. EAE model: animals were dosed on days −3, 0, 3 and 6. **F** Disease score in murine EAE model (*n* = 12), statistics shown for *HSA-IL2m* against both PBS and *HSA* groups (Kruskal–Wallis). **G** Frequency of Tregs (FOXP3+) among CD4 T cells in spleen and inguinal lymph nodes (*n* = 8). **H** Frequency of MOG-specific (Tetramer+, Supplementary Fig. 15) Th1 (T-bet+) cells in spleen and inguinal lymph nodes (*n* = 8). **I** Frequency of IFNγ expression in splenic antigen experienced CD4 T cells (*n* = 8). Asterisks denote significance (two-way ANOVA, two-sided, *p*-value * < 0.05, ** < 0.01, *** < 0.001). Box plots center is the median, bounds of box are 1st and 3rd quartile. Upper whisker is the largest value that is no greater than 1.5× the interquartile range plus the third quartile. Lower whisker is the smallest value that is no smaller than the first quartile minus 1.5× the interquartile range. Source data are provided as a Source Data file.

Having demonstrated selective activation and expansion of Tregs with *HSA-IL2m*, we next sought to evaluate its activity in preclinical disease models of inflammation. We chose an acute model of graft vs host disease, which is initiated by the transfer of B6 splenocytes into B6 x DBA F1 mice. This results in donor T cell activation in response to recognition of host allogeneic antigens, acquisition of donor CTL activity, and the elimination of host lymphocytes 10–14 days after transfer. We hypothesized that the expansion and activation of Tregs could prevent the activation and proliferation of the donor CD8+ T cells. To test this, we treated recipients with PBS, *HSA-IL2m* or an *HSA* control mRNA, and non-transfer naïve (NA) host mice served as baseline controls. Mice treated with PBS and *HSA* lost weight 10 days after donor cells transfer (Fig. 6A). In contrast, mice treated with *HSA-IL2m* maintained body weight, similar to the naïve mice. In order to assess the molecular and cellular changes associated with this reduction in disease severity, we analyzed splenocytes and PBMCs 14 days after cell transfer. Host B cells were depleted in both spleen and blood of *HSA* and PBS animals (Supplementary Fig. 13A), and the remaining cells are primarily from donor (Fig. 6B). Donor CD8 T cells were increased in numbers in the *HSA* and PBS treated groups (Supplementary Fig. 13A), stemming from expansion of transferred donor cells (Fig. 6C). In contrast, treatment with *HSA-IL2m* maintained the pool of host B cells, suppressed donor CD8 T cell engraftment, and increased the number of Tregs (Supplementary Fig. 13A). We extracted a variety of parameters characterizing the phenotype of T and B cells from our flow cytometry data and performed a principal component analysis. We observed that *HSA-IL2m* and NA animals clustered together, separated along dimension 1 compared to the *HSA* and PBS treated animals (Supplementary Fig. 13B). Contributions to dimension 1 were driven by the frequency of host/donor cells and activation of CD8+ T cells. Indeed, in naïve mice, CD8 T cells were primarily negative for T-bet, GZMB and PD-1 (Supplementary Fig. 13C). However, following cell transfer and treatment with *HSA* or PBS, donor CD8 T cells were all PD-1+ in blood and in majority in the spleen. Furthermore, more than 60% of donor CD8 T cells co-expressed T-bet, GZMB and PD-1. In contrast, *HSA-IL2m* treatment resulted in CD8 T cells that were phenotypically similar to those of naïve animals. Functionally, cells from mice receiving different treatments were distinct in their capacity to produce IFNγ. Indeed, the percentage of IFNγ producing cells were comparable in *HSA-IL2m*-treated and naïve animals but increased in donor CD8 T cells from the *HSA* and PBS treatment groups (Fig. 6D). Finally, the frequency of IFNγ producing CD8 T cells was inversely correlated with the frequency of Tregs (Fig. 6E). Inflammation reduction with *HSA-IL2m* treatment was also reflected in the serum concentration of cytokines such as TNFα and IFNγ (Supplementary Fig. 13D). Taken together, these results suggest that expansion of Tregs can prevent the engraftment and activation of donor CD8 T cells, along with the subsequent depletion of host lymphocytes in this model of acute graft-vs host disease.

We sought to validate the efficacy of Treg expansion in a second disease model. We chose the myelin oligodendrocyte glycoprotein (MOG) experimental autoimmune encephalomyelitis model, in which tolerance to a myelin antigen is broken via immunization of adjuvanted MOG35-55 peptide. Approximately 10 days after immunization, animals started to develop signs of mobility impairment (disease onset), which was significantly delayed (*p*-value = 0.034) by treatment with *HSA-IL2m* (Fig. 6F). Total disease burden, as measured by area under the curve was also reduced (*p*-value = 0.031). To explore potential mechanisms of protection, we isolated cells from blood, draining (inguinal) lymph nodes and spleen at 10 days. The frequency of regulatory T cells was elevated in spleens (Fig. 6H). The frequency of MOG35-55 specific Th1 cells was greatly reduced in inguinal lymph nodes and spleen of *HSA-IL2m* treated animals compared to the PBS and *HSA* controls (Fig. 6H). Furthermore, *HSA-IL2m* reduced the fraction of IFNγ producing CD44hi (antigen-experienced) conventional CD4 T cells (Fig. 6I). To investigate the effects of *HSA-IL2m* at the site of central nervous system inflammation, we performed a transcriptomic analysis of spinal cord tissue (Supplementary Fig. 14). Ten days post immunization, the *HSA-IL2m*-treated animals had several downregulated genes associated with trafficking of immune cells (*Sell*, *Cybb*, *H2-Aa*, *H2-Ab1*, and *H2-Eb1*). This reduction in cell trafficking was consistent with the reduced immune cell chemotactic factor signature (*Ccl2*, *Cxcl10*, *Ccl5*, *Ccl19*, and *Ccl7*). Decreased inflammation was also reflected in the diminished expression of antigen presentation genes (*Cd74*) and tissue damage sensing genes (*Zbp1*). Overall, these observations are consistent with a reduction in the cycle of tissue damage, activation of macrophages, secretion of recruitment factors, infiltration of immune cells and additional tissue damage.

## Discussion

In this study, we set out to improve the pharmacology of IL-2 to achieve sustained and specific Treg activation. To do so, we introduced mutations to IL-2 to promote the differential affinity to the dimeric and trimeric IL-2 receptors and fused it to human serum albumin to confer prolonged serum half-life. This construct can selectively activate human Tregs in vitro. In vivo, injection of lipid nanoparticle formulated *HSA-IL2m* mRNA resulted in the expression of HSA-IL2m, which was detected in plasma. We found that the protein produced by *HSA-IL2m* had a longer plasma half-life than *HSA-IL2wt* encoded protein in both mouse and cynomolgus monkey. The short half-life of unmodified IL-2^38^ is generally attributed to its size and consumption by IL-2R expressing cells. Using NOD.*scid* il2ra^null^ mice, previous work reported that IL-2 half-life is strongly influenced by the expression of IL2RA^39^. Thus, we hypothesize that the prolonged half-life of HSA-IL2m compared to HSA-IL2wt stems from the reduced consumption by Tcons and NK cells.

In mice and cynomolgus monkey treated with *HSA-IL2m*, we observed a dose-dependent expansion and activation of Tregs, and absence of the activated NK cell, CD8 T cell and release of pro-inflammatory plasma cytokines traditionally associated with IL-2 therapy^40^. Using single cell RNA sequencing, we identify 6 distinct Treg populations, two states associated to ST2 Tregs, one Tfr population, and three states of Tregs, which had different dependencies on IL-2 signaling. In naive mice, the dominant Treg state in the spleen was OxTreg. Tregs rely on fatty acid oxidation (FAO) and oxidative phosphorylation^41^ and IL-2 reinforces this state in part through activation of Raptor/mTORC1 signaling^42^. In addition, FOXP3 also has been reported to enhance the transcription of genes of the electron transport system^43^. Why oxidative phosphorylation is the preferred metabolic state of Tregs is unclear, but several reports demonstrate its importance for Treg function^44^. Genetic ablation of mitochondrial complex III in Tregs leads to a scurfy-like disease with normal Treg number but reduced suppressive ability^45^. Similarly, upregulation of Treg glycolysis via the overexpression of GLUT1 leads to expanded numbers but reduced functionality and stability, culminating in spontaneous autoimmunity^46^. These reports and others suggest that glycolysis might be activated in order to fuel the proliferation and migration^47^ of Tregs, but detrimental to the suppressive effects. Although Tregs have increased glycolysis and FAO during proliferation, in our data, the two mitotic Tregs topics did not have differentially expressed genes associated with glycolysis. Yet, they had reduced OxPhos related genes compared to their counterpart non-mitotic Tregs. Oxidative phosphorylation favors the production of reactive oxygen species and ATP. One hypothesis is that Treg favor OxPhos in order to fuel the generation of immunosuppressive adenosine, which is converted from ATP by ectoenzymes CD39 and CD73^48–50^.

Tfr cells represented a large proportion of Tregs at baseline. They are involved in the regulation of B cell responses through repression of excessive follicular helper T cells^51^ (Tfh) and help to B cell via IL-10 production^52^. This population was insensitive to treatments. In fact, like Tfh, Tfr have been reported to be negatively regulated by IL-2 signaling. IL-2 deficient mice have reduced Treg numbers but a large proportion of Tfr, and treatment with IL-2/anti-IL-2 complexes diminished the Tfr pool^53^. In a murine model of influenza infection, IL-2 was shown to inhibit Tfr development via inhibition of Bcl-6 and upregulation of Blimp-1^54^. Furthermore, these authors demonstrated that Tfr derived from CD25+ Tregs. These observations have important significance for the development of Treg expanding therapies since Tfr control antibody responses^53^. Therefore, reducing these population via IL-2 treatment could be detrimental. Muteins that have increased specificity for the high affinity receptors could thus offer a therapeutic advantage by preserving Tfr while expanding and activating Tregs.

ST2 Tregs reside primarily in non-lymphoid tissues and are traditionally expanded by IL-33. The two topics we assigned to ST2 Tregs were characterized by the expression of *Il13*, *Il1rl*, *s100a6*, *Lgals1*, *S100a2*, *Klrg1*, *Gzmb*, *Ass1,* and *Tgln2*. This signature was in accordance with that of a population previously reported to expand with a murine IL-2 mutein^55^. This subset plays an important role in limiting inflammation and promoting tissue repair^35,56,57^. ST2−/− Treg adoptive transfer failed to suppress colitis in a murine adoptive transfer model, demonstrating that ST2 signaling on colonic Tregs is important for colonic Treg function and adaptation^57^. Two precursor stages of ST2 Treg have been reported in the spleen and lymph nodes via scRNAseq^58^ and BATF was identified as a key driver of the ST2 Treg program. In our data, *Batf* was differentially upregulated in the ST2 Treg topic. ST2 Tregs in the spleen were expanded and activated to a greater degree with *HSA-IL2wt* that with *HSA-IL2m*, but the difference could be surmounted by an increased dose of *HSA-IL2m*. Thus, this subset requires higher levels of IL-2 signaling to proliferate and/or differentiate^59^. This is an important implication to Treg expanding therapies as they have so far focused on the expansion of Tregs as a group, and not investigated individual populations. Our data indicate that the dynamics of Tregs and their suppressive potential can differ in ways that conventional quantification methods can fail to capture. Dosing selection could be strongly influenced by the differential expansion of Treg subsets with distinct tissue abundance and specialization.

Beyond effects on Tregs, *HSA-IL2m* dramatically reduced the frequency of a subset we labeled as aTeff. This topic upregulated *Uba52* and many ribosomal genes. T cells rapidly upregulate genes involved in the translation machinery upon activation^60^. In contrast the naïve T cell topic increased in proportion with *HSA-IL2m* treatment. Thus, we hypothesize that these cells switched from a highest probability topic of aTeff to T_N_ indicating an increase in their activation threshold. Deficiency in FOXP3 has cell extrinsic effect on conventional T cells^61^, so we hypothesize that in a similar fashion, increased Treg levels in the absence of IFN stimulation achieved with *HSA-IL2m* can alter the state of conventional T cells and raise their activation threshold.

To assess whether the expansion and activation of Tregs could confer efficacy in preclinical models, we evaluated our formulated *HSA-IL2m* mRNA into two murine models. In the murine acute graft-versus-host model, polyclonal activation of donor cells results in activation and expansion of donor CD8 cells and acquisition of markers of cytolytic function (e.g., GZMB and IFNγ), which are responsible for loss of host immune cells (B cells, CD4 and CD8 T cells). In parallel with Treg expansion, we observed preservation of host body weight and host B cells, and little or no donor CD8 T cell expansion with greatly reduced acquisition of CD8 cytolytic markers in those donor cells that were present. This indicates that *HSA-IL2m* LNP treatment can induce functional Tregs capable of controlling a widespread self-reactive T cell driven disease model. In a similar manner, the MOG peptide model of EAE is widely used to assess drug candidates for multiple sclerosis and serves as a mechanistic model for activation and expansion of self-reactive T cells (Th1, Th17) that induce and propagate injury to the spinal cord of mice. We observed a delay in disease onset and reduced severity of clinical symptoms, corresponding to the expansion of Tregs and reduced expression of proinflammatory genes in spinal cord tissue. Through these two models, *HSA-IL2m* was demonstrated to be efficacious in expanding Tregs and ameliorating clinical severity in preclinical models of T-cell-mediated human autoimmune diseases.

There has been growing evidence that increasing the number of Tregs could confer clinical benefits to patients with autoimmune and inflammatory disorders^15,62^. Thus, formulated *HSA-IL2m* treatment of patients with autoimmune diseases may provide benefit through the preferential expansion and activation of Tregs, which could suppress pathogenic autoreactive effector T cells. This next generation immunotherapy has the potential to restore the balance between Tregs and effector cells that is perturbed in autoimmune disease and chronic inflammatory disorder. Moreover, the use of mRNA technology enables further innovative therapeutics through effortless combination therapies^29^, an approach that has been increasing in popularity in many immune-related indications^63–65^. The demonstration of biological activity on human T cells combined with preclinical proof of ameliorated T cell-mediated autoimmunity by *HSA-IL2m* warrants clinical investigation.

## Methods

### mRNA synthesis and formulation

*HSA-IL2wt* and *HSA-IL2m* mRNA, encoded either the homo sapiens IL-2 wild-type or a mutant fused to Human Serum Albumin, to extend protein plasma half-life. *HSA-IL2m* contained a N88D^39,66,67^ mutation to destabilize IL2RB interaction by preventing a hydrogen bond with an Arginine 42 in IL2RB, and V69A, Q74P^68^ mutations to improve IL2RA and reduce IL2RB affinity. The resulting constructs were synthesized by in vitro transcription (IVT). A plasmid encoding the T7 RNA polymerase promoter followed by 5′ untranslated region (UTR), open reading frame (ORF), 3′UTR, and polyA tail was overexpressed in *E. coli*, linearized, and purified to homogeneity. The mRNA was synthesized by using a modified T7 RNA polymerase IVT, where uridine triphosphate was substituted completely with N1-methyl-pseudouridine triphosphate^69^. Cap 1 was utilized to improve translation efficiency. After the transcription reaction, mRNAs were purified, buffer exchanged into sodium citrate, and stored at −20 °C until use. Formulation of mRNA were performed as described previously^70^. Analytical characterization assays included particle size and polydispersity, encapsulation, and endotoxin content which had to meet predefined specifications before the material was deemed acceptable for in vivo study. Final particle size and encapsulation were <100 nm and >80%, respectively, with endotoxin below 10 EU/mL.

### HSA-IL2m and HSA-IL2wt recombinant protein expression and purification

Plasmids encoding for HSA-IL2wt and HSA-IL2m were transfected into Expi293F cells and the cells were incubated for 6 days at 37 C, 8% CO_2_, and 55 RPM rocking speed. Supernatants were harvested and dialyzed into 20 mM Sodium Phosphate, pH 7.0. The solution was purified through a HiTrap Blue Sepharose column and eluted using a sodium chloride gradient. The resulting material was then concentrated via a 10 kDa molecular weight cut-off spin column and purified by size exclusion chromatography on a Superdex 200 column. The final material was assessed for purity by SDS-PAGE and HPLC

### In vitro STAT5 phosphorylation assay

Cells were incubated in RPMI 1640 + 10% FBS for the indicated time at the indicated concentration of IL-2 protein, in a 37 C, 5% CO_2_ incubator. Immediately after, cells were placed on ice, spun down at 500 × *g* for 5 min and resuspended in PBS + 2% FBS + 1 mM EDTA + eFluor780 viability dye (eBiosciences #65-0865-14). Cells were incubated on ice for 10 min. Cells were then fixed and permeabilized using the Biolegend True Nuclear kit (#424401). The cells were then incubated with fluorophore-conjugated antibodies in permeabilization buffer. The panel of specific antibodies used are detailed in Table S1. After overnight incubation, the cells were washed and analyzed on a BD Fortessa cytometer.

### Animals

C57BL/6 female mice (unless specified otherwise) were purchased from Charles River Labs (#002014) or Jackson (#000664)). Rag1^−/−^ (#RAGN12) were purchased from Taconic. FOXP3-GFP (#006772) mice were purchased from Jackson Labs. DBAxB6 F1 mice were purchased from Charles River Labs (#099). All mice strains were 8 weeks old at the experimental start. Cynomolgus male monkeys between the ages of 2 and 4 years old were housed at a Charles River facility. The animal facility is specific pathogen-free and the control animals were co-housed or held in the same holding room. Mice were euthanized by carbon dioxide asphyxia. All animal experiments were performed in accordance with federal, state, local and institutional IACUC policies. These policies are determined by the USDA and are further enforced by the MSPCA and Cambridge Commission. Moderna is regularly monitored to ensure compliance to these rules.

### Immunophenotyping by flow cytometry

Organs were mechanically disrupted using a sterile syringe plunger through a 70 μm nylon strainer (Falcon, Catalog #352350) to obtain single cell suspensions. Single cell suspensions were resuspended in 50 μL of surface antibody staining solution, diluted in PBS + 2% FBS + 1 mM EDTA (PBSFE), and incubated for 60 min at 4 °C on a shaking platform. Cells were washed, fixed and permeabilized. Finally, cells were stained with the intracellular target antibodies overnight (12–16 h) in antibody solution diluted in permeabilization buffer (Biolegend, Catalog #424401). For intracellular cytokine detection, single cell suspensions were resuspended in 100 μL of RPMI1640 + 5% FBS and PMA/ionomycin activation cocktail (Biolegend, Catalog # 423302) and incubated at 37 °C, 5% CO_2_. After 1 h, 10 μL of a 10× solution of Brefeldin A (Biolegend, Catalog #420601) and Monensin (Biolegend, Catalog #420701) diluted in RPMI1640 + 5% FBS were added and cells were incubated for an additional 4 h. Next, cells were stained intracellularly as described above. Samples were acquired on a BD Fortessa flow cytometer. Antibodies were purchased from Biolegend and BD Biosciences and were used at optimized dilutions. Illustrative flow cytometry gating is show in Supplementary Fig. 16. Antibody-fluorophore combinations, clones and dilution are reported in Table S2.

### Treg suppression assay

The spleens from CD45.2 FOXP3-GFP mice were harvested 4 days after treatment with LNP. The CD4 T cells were isolated using a magnetic bead negative selection kit from StemCell. The enriched CD4 T cells were then sorted based on GFP expression. In parallel, CD45.1 conventional T cells were isolated by a magnetic negative selection kit from StemCell, and the cells were labeled with Cell Trace Violet (CTV). The CD45.2 GFP Tregs and the CTV-labeled CD45.1 conventional T cells were incubated at various ratios for 3 days in the presence of 30 K splenocytes from Rag1^−/−^ mice and 0.3 μg/mL anti-CD3 antibody. After 3 days in culture, the cells were stained and analyzed by flow cytometry for CTV peak dilutions in the CD45.1 population.

### EdU incorporation proliferation assay

Single cells suspensions from spleens were prepared as described above. Samples were incubated in RPMI 1640 + 10% FBS at 37 °C for 2 h in the presence of Click-it EdU reagent (ThermoFisher #C10419) at a 1:1000 dilution. The remainder of the procedure was followed as per the manufacturer’s recommendations. Cells were stained for 1 h with viability dye eFluor780 (ThermoFischer L34961) at 1:100 dilution, and surface antibodies CD3e AF488 (1:400, Biolegend 100321), CD4 BV510 (1:1600, Biolegend 100449), CD8a BV570 (1:400, Biolegend 100740), NK1.1 AF700 (1:800, Biolegend 108730), CD25 BV421 (1:400, Biolegend 102043). Samples were acquired on a Cytek Aurora flow cytometry instrument.

### Spinal cord transcriptomics

RNA was isolated from spinal cord tissue using automated Promega Maxwells with the RSC simplyRNA Tissue Kit as per the manufacturer’s recommendations (Promega, cat #AS1340). RNA quality was assessed using Agilent Tape Station RNA ScreenTape (Agilent, cat #5067-5576) and quantitated by Invitrogen Quant-iT RNA Broad Range Assay Kit (Invitrogen, cat #Q10213). 150 ng of RNA was used as input for NanoString Autoimmune Panel (NanoString cat #XT-CSO-MAIP1-12) according to nCounter XT CodeSet Gene Expression Assays protocol. Resulting RCC files were examined with NanoString nSolver software for QC analysis. Briefly, samples had a background threshold set to 25 counts and normalized to a set of housekeeping genes with low variance (%CV < 40%) and medium or high counts (>50).

### scRNA-seq experiment

Spleens were collected from C57BL/6 female mice dosed with *HSA-IL2wt* or *HSA-IL2m* at 0.05mpk at 2 days and 4 days post injection (*n* = 3 mice each condition and timepoints) and from untreated mice for baseline comparison (*n* = 2). Each spleen was homogenized by mechanical disruption and CD4 T cells were isolated using a negative selection magnetic enrichment kit (StemCell). CD4 T cells collected from each treatment condition were pooled across individual mice, resulting in five single-cell suspensions. For each sample, the purity exceeded 98% purity and viability exceeded 99%. Each sample was diluted to a 1 million cells per mL.

### scRNA-seq library preparation and sequencing

10× Genomics 3’ Library and Gel Bead Kit (Chromium Next GEM v3.1 1000128, 1000157, and 1000213) and Single Cell Chip G kit (Chromium Next GEM 1000127) were used to prepare libraries using the 10× Genomics Chromium Controller according to the manufacture’s guidelines (CG00052 Rev D). Two chips were used with a target 10,000 cells collected in each lane. The five pooled samples were each split into technical replicates and distributed to the two chips as follows: The untreated pooled sample was split into four technical replicates with two replicates distributed to each chip. For the treated conditions, each pooled sample was split into three technical replicates, with one replicate from Day 2 and two replicates from Day 4 on Chip 1, and two replicates from Day 2 and one replicate from day 4 on Chip 2.

All libraries were sequenced together in one run on Illumina NovaSeq6000 Platform. bustools (v0.39.3)^71^ was used to map sequence reads to mm10 genome (https://www.gencodegenes.org/mouse/release_M16), as well as to perform demultiplexing, barcode processing and gene count.

### scRNA-seq data analysis workflow

R/Bioconductor workflows^72^ were used as the primary tools for data analysis (R v4.1.2^73^, Bioconductor v3.13^74^). Python package scanpy (v1.7.2)^75^ module PAGA was used to reconstruct Treg subtype associations.

### scRNA-seq data preprocessing and quality control

DropletUtils(v1.14.2)^76^ EmptyDrops function was used to distinguish droplets containing cells and droplets containing cell-free RNA (FDR threshold < .01, niter = 100,000 for the Monte Carlo *p*-value calculation). Cells were further excluded if they were associated with low number of molecules (<500 total molecules) or low number of genes detected (<500 genes). Low count genes were excluded to retain genes detected in >0.01% of the cells. The result is 164,240 single-cell gene expression profiles: 27,852 cells in Untreated, 33,544 cells in *HSA-IL2wt* Day 2, 24,733 cells in *HSA-IL2wt* Day 4, 37,100 cells in *HSA-IL2m* Day 2, 41,011 cells in *HSA-IL2m* Day 4.

### scRNA-seq topic modeling

We applied topic model^77^, also known as grades of membership model^34,78,79^, to the count matrix of cells that passed our quality control. The method does not require a data preprocessing step in which the UMI counts are log-transformed and normalized. Fitting of the topic model was performed using the fastTopics package (v0.5-25)^80^, which is a successor to the CountClust Bioconductor package (v1.18.0)^34^. fastTopics implements fast and scalable algorithms for fitting topic models by exploiting the special relationship between the multinomial topic model and Poisson non-negative matrix factorization.

### scRNA-seq differential expression analysis to identify topic-defining genes

Differential expression analysis was performed via fastTopic diff_count_analysis function, which is designed for identifying genes defining or characterizing each topic. fastTopic’s approach to differential expression analysis is different from conventional differential expression analysis^81^. In conventional differential expression analysis, cluster membership is assumed to be dichotomous for each cell (either assigned to a cluster or not). While, in topic modeling, each cell is allowed partial membership in different cluster, and these partial membership weights sum up to 1 across topics for each cell. fastTopics diff_count_analysis computes log-fold-changes (LFC) statistics, considering the partial memberships across topics. For a given topic k, LFC is defined as the log_2_ ratio of the mean UMI count for all cells belonging to topic k over the mean UMI count among cells not belonging to the topic^80^. Finally, we ranked genes by LFC and selected the top 100 in the list as the topic-defining gene for each topic.

### scRNA-seq Normalization

Log2-normalized counts were generated and used as input for differential state analysis, gene set enrichment analysis, cell cycle stage assignment, and for visualizing single-cell expression profiles in UMAP embeddings and mean gene expression profiles. scran (v1.20.1)^82^ calculateSumFactors function was used to compute normalized library size factors (min.mean = 0.1 to use only genes with mean UMI count >0.1).

### scRNA-seq gene set enrichment analysis

We performed gene set enrichment analysis on the CD4 T cell families to characterize enriched pathways in. Mouse Hallmark collection gene sets in the Molecular Signatures Database (MSigDB v7.4)^83,84^ were downloaded using CRAN msigdbr (v7.2.1). scran (v1.20.1)^82^ findMarkers function^72^ was applied to implement Welch *t*-test between pairs of clusters and compute LFC. The minimum of LFC was chosen to represent the distance between a given cluster and every other cluster and used as input for enrichment analysis. Bioconductor package fgsea (v3.12)^85^ fgseaSimple function was used to implement the gene set enrichment analysis (minimal and maximal of a gene set to test is 5 and 500, respectively, 200,000 permutations done to set a low nominal *p*-value). False discover rate threshold was set at 0.05.

### scRNA-seq differential state analysis to compare IL-2 responsivity

We applied edgeR (v3.34.1)^86,87^ to detect treatment-specific state transition in gene expression profile at the sample replicate level. Prior to applying edgeR, gene expression data were pooled and aggregated across cells within each sample replicate for each topic (*n* = 4 in Untreated, and *n* = 3 in WT_2, WT_4, M_2, M_4) using scuttle (v1.2.1)^88^ aggregateAcrossCells function. The edgeR function filterByExpr was used to retain genes with sufficient sample size, resulting in 10,094 genes for the analysis. We fit the model of log2 normalized count ~ treatment condition (Untreated, WT_2, WT_4, M_2, M_4) + Topic (16 topics) and used the limma (v3.48.3) function voom^89^ to estimate the mean-variance relationship. False discovery threshold was set at .05.

### scRNA-seq cell cycle stage assignment

cyclone (scran v1.20.1)^36^ was used to assign each single cell to a discrete cell cycle stage (G1, S, G2.M). The method builds a training data based on user-input reference dataset and assigns each single cell to a cell cycle stage. To build the training data, the method computes the sign of the difference in expression between each pair of cell cycle genes. Pairs with changes in the sign across cell cycle stages are chosen as markers. In the assignment stage, each single cell is assigned to the different stages based on whether the observed sign for each marker gene pair is consistent with the sign in the training data. We used one of the cyclone reference mouse single-cell RNA-seq data set to build our training data. cyclone was implemented using the cyclone function in the Bioconductor scran package (v1.20.1).

### scRNA-seq partition-based graph abstraction to reconstruct Treg association

We used scanpy (v1.7.2)^90^ module PAGA to reconstruct associations between Treg subtypes. Raw count expression matrix of 24,022 cells and 18,840 genes assigned to Treg family were preprocessed using Scanpy normalization and filtering pipeline. 999 genes were retained for downstream analysis. Principle Component Analysis was applied to the 99 genes, and the top 20 principal components were used to construct a symmetric kNN-like graph (*n* = 30 cells in the neighborhood) using the approximate near neighbor search within UMAP (each node represents a Treg topic). Based on the pre-assigned Treg topics, an undirected graph was generated using a statistic quantifying the degree of connectivity of two clusters. The edges with connectivity threshold >0.2 was retained in the data to define a Treg association graph.

### Software

For single cell RNA sequencing, all analyses were run in R v4.1.2^73^ with Bioconductor v3.13^74^. For flow cytometry data, analyses were run in R v4.1.2 and in FlowJo version 10.

### Acute GvHD model

Spleen and lymph nodes from C57BL/6 mice were harvested and prepared as a single cell suspension. Each recipient DBA x B6 F1 mice received 50 million C57BL/6 cells by tail vein injection on day 0. Formulated mRNA was injected subcutaneously at 0.1 mpk on day −3, 0, +3, +7, and +10. Blood was collected on day 10 and day 18. Animals were euthanized and spleens were isolated on day 18.

### EAE model

C57BL/6 female mice received a subcutaneous injection of 100μg of MOG35-55 peptide emulsified with CFA and 2.5 mg/mL *M. Tuberculosis H37Ra*. In addition, animals received an intraperitoneal injection of 0.01 mg/kg of Pertussis toxin and a second dose two days later. Formulated mRNA was injected subcutaneously at 0.1 mpk on day −3, 0, +3, and +6. Starting on day 10, animals were weighed daily and scored based on the following system; 0: normal, no overt signs of disease, 1: tail paresis, 2: righting reflex impaired, 3: partial hind limb paralysis, 4: complete hind limb paralysis or absence of ambulation, 5: complete hind limb paralysis with front limb paresis. 10 or 18 days after immunization, blood, spleen, inguinal lymph nodes and spinal cords were isolated.

### Statistics

For the EAE model, to compare rates of disease-free progression between treated and control groups, a two-sided log-rank survival test was performed. Bonferroni correction using a family-wise significance level of 5% was used to determine pairwise significance. For the day of onset and the clinical score curve AUC (area under the curve), comparisons between treatment and controls were performed using a Kruskal–Wallis non-parametric test. For the immunophenotyping and plasma concentration continuous variables, one-way ANOVAs were performed. If the overall F-test from the one-way ANOVA was significant, Tukey’s pairwise comparison tests was performed between groups as appropriate. For the Nanostring data, fold changes were computed, and *p*-values were adjusted using the Benjamini–Yekutieli multiple comparison correction. All statistical tests were two-tailed and *p*-values < 0.05 were considered statistically significant.

### Reporting summary

Further information on research design is available in the Nature Research Reporting Summary linked to this article.

## Supplementary information

Supplementary Information

Reporting Summary

## Data Availability

The data that support the findings of this study are available from the corresponding author upon reasonable request. The raw numbers charts and graphs are available in the Source Data file whenever possible. Single-cell RNA-seq data generated in this study have been deposited in the GEO database under the access code GSE200142. Source data are provided with this paper.

## Source data

Source Data

## Code availability

The code and processed data associated with single-cell RNA-seq data analysis are available at GitHub: https://github.com/modernatx/selective_activation_expansion_Treg_using_lipid_encapsulated_mRNA_encoding_longacting_IL2_mutein.
