# Supplementary material for: Selective activation and expansion of regulatory T cells using lipid encapsulated mRNA encoding a long-acting IL-2 mutein
Source: Nat Commun. 2022 Jul 5;13:3866. doi: 10.1038/s41467-022-31130-9 (PMC9256694; doi:10.1038/s41467-022-31130-9)
Supplement: Supplementary file 1 — Supplementary Information [file 41467_2022_31130_MOESM1_ESM.pdf]

Supplementary Information

Selective activation and expansion of regulatory T cells using lipid encapsulated mRNA encoding a long-acting IL-2 mutein.

Seymour de Picciotto[1,*], Nicholas DeVita[1], Chiaowen Joyce Hsiao[1], Christopher Honan[1], Sze-Wah Tse[1], Mychael Nguyen[1], Joseph D. Ferrari[1], Wei Zheng[1], Brian T. Wipke[1], Eric Huang[1,*]

[1]Moderna, Inc., Cambridge, MA, 02139, USA

1. Supplementary Figures

Supplementary Figure 1:

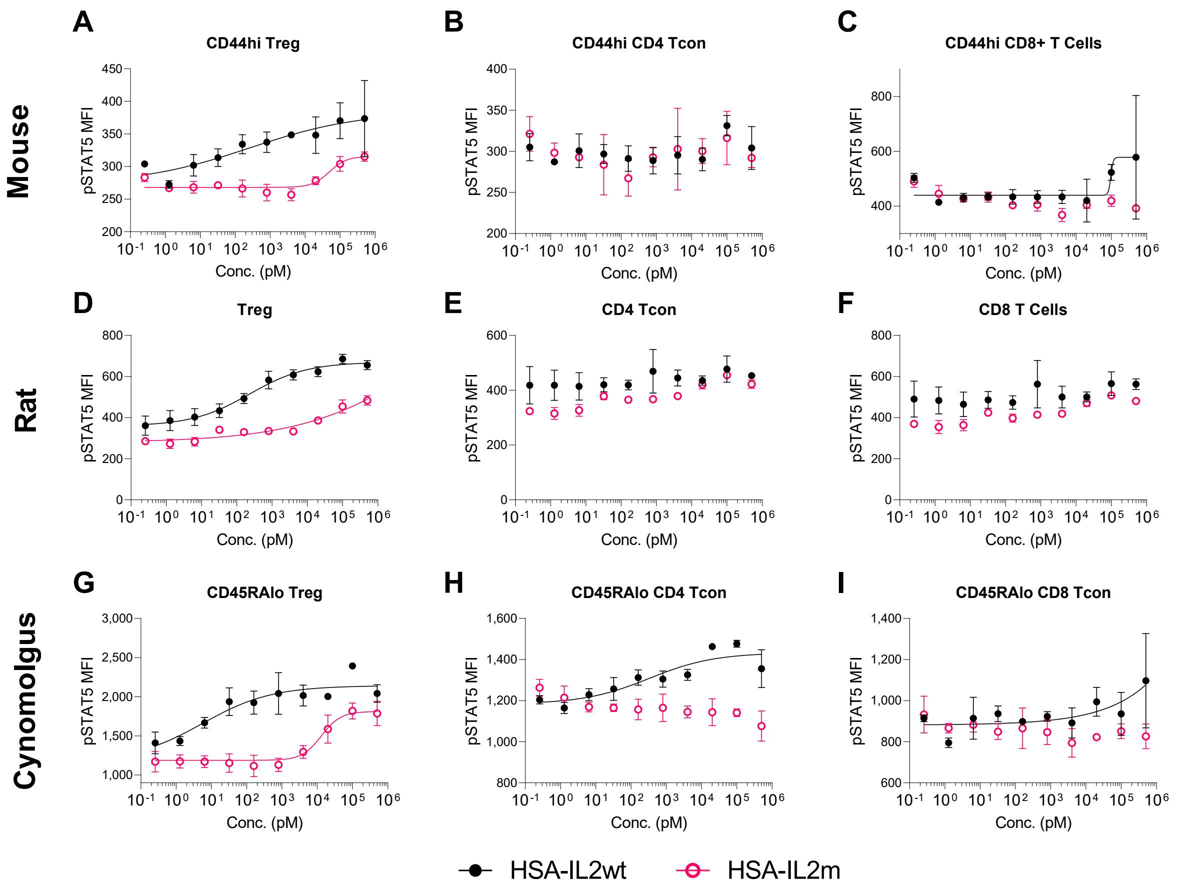

***In vitro* characterization on mouse, rat and cynomolgus monkey T cells of HSA-IL2wt and HSA-IL2m induced STAT5 phosphorylation**. HSA-IL2wt and HSA-IL2m protein titrations on murine **A)** memory Tregs, **B)** memory CD4+ conventional T cells, and **C)** memory CD8+ T cells after 30-minute incubation. HSA-IL2wt and HSA-IL2m titrations on rat **D)** memory Tregs, **E)** memory CD4+ conventional T cells, and **F)** memory CD8+ T cells after 30-minute incubation. HSA-IL2wt and HSA-IL2m titrations on cynomolgus monkey **G)** memory Tregs, **H)** memory CD4+ conventional T cells, and **I)** memory CD8+ T cells after 30-minute incubation. Data are presented as mean ± s.d, fits are derived from log-logisitic model, n=4.

Supplementary Figure 2:

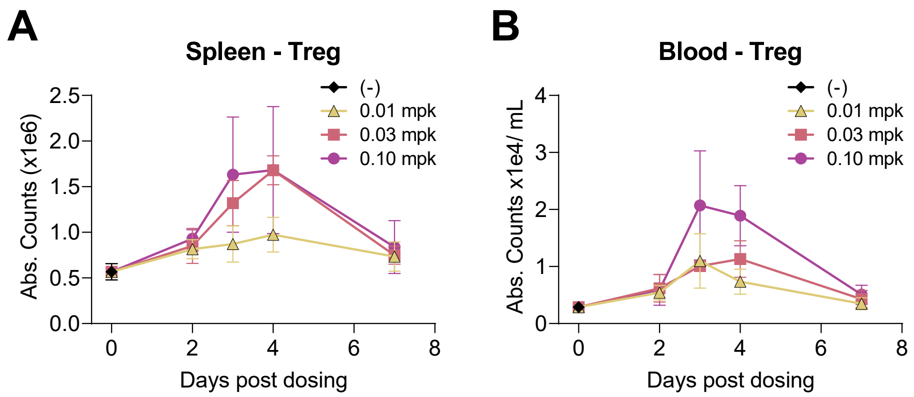

Kinetics of Treg expansion in C57BL/6 with *HSA-IL2wt*. Murine Treg counts were determined in the A) spleen and B) blood following subcutaneous administration of *HSA-IL2wt* at 0.01, 0.03 and 0.1 mpk (n=6). Data shown as mean ± s.d.

Supplementary Figure 3:

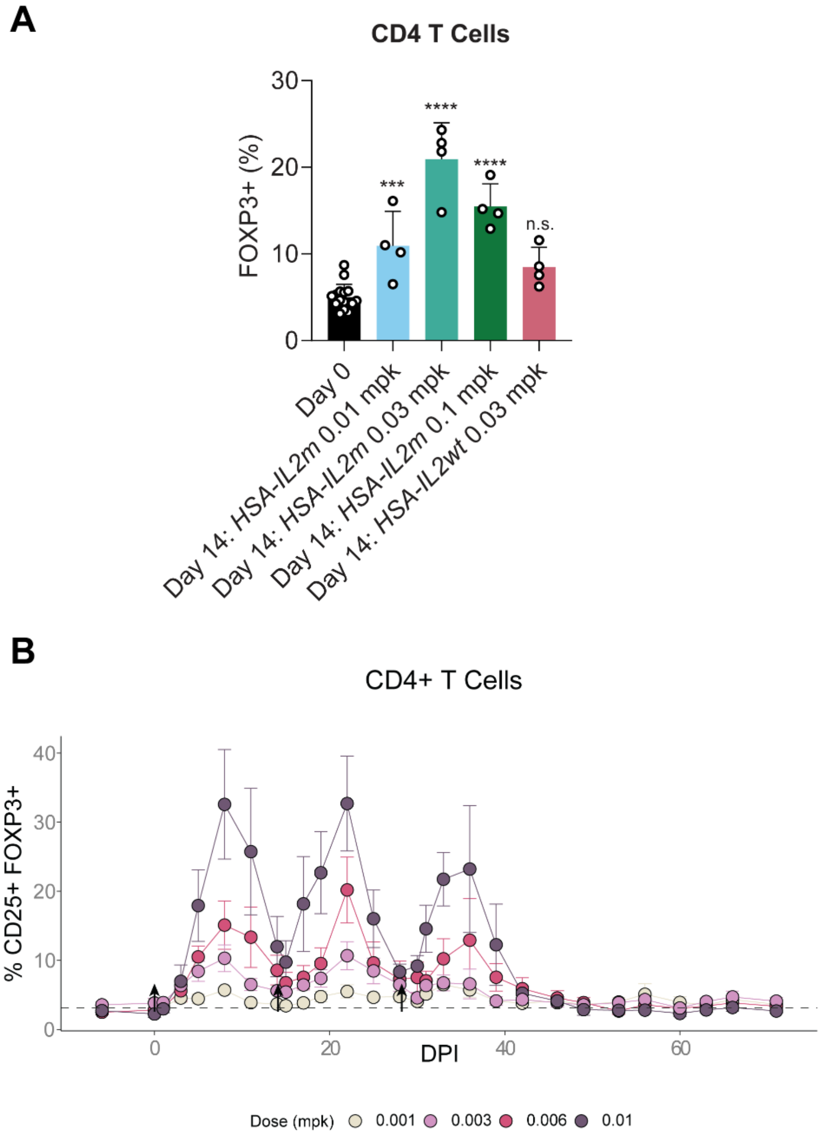

Kinetics of Treg expansion with *HSA-IL2wt* and *HSA-IL2m* in cynomolgus monkey. (A) Frequency of Treg populations in cynomolgus blood as a function of *HSA-IL2m* or *HSA-IL2wt* dose. Data presented as mean ± s.d, n = 16 for Day 0, n = 4 for all others. Asterisks denote significance (One-way ANOVA, p-value *** < 0.001, **** <1e-4; n.s. = not significant) compared to baseline (Day 0). Exact P-values, from left to right: P = 9e-4, <1e-4, <1e-4, 7.24e-2. (B) Frequency of Tregs among CD4 T cells in cynomolgus monkey following repeat administration

of *HSA-IL2m*. Arrows indicate time on injection. Data are presented as mean ± s.e, n = 4.

Abbreviations: DPI = Days Post first Injection.

Supplementary Figure 4:

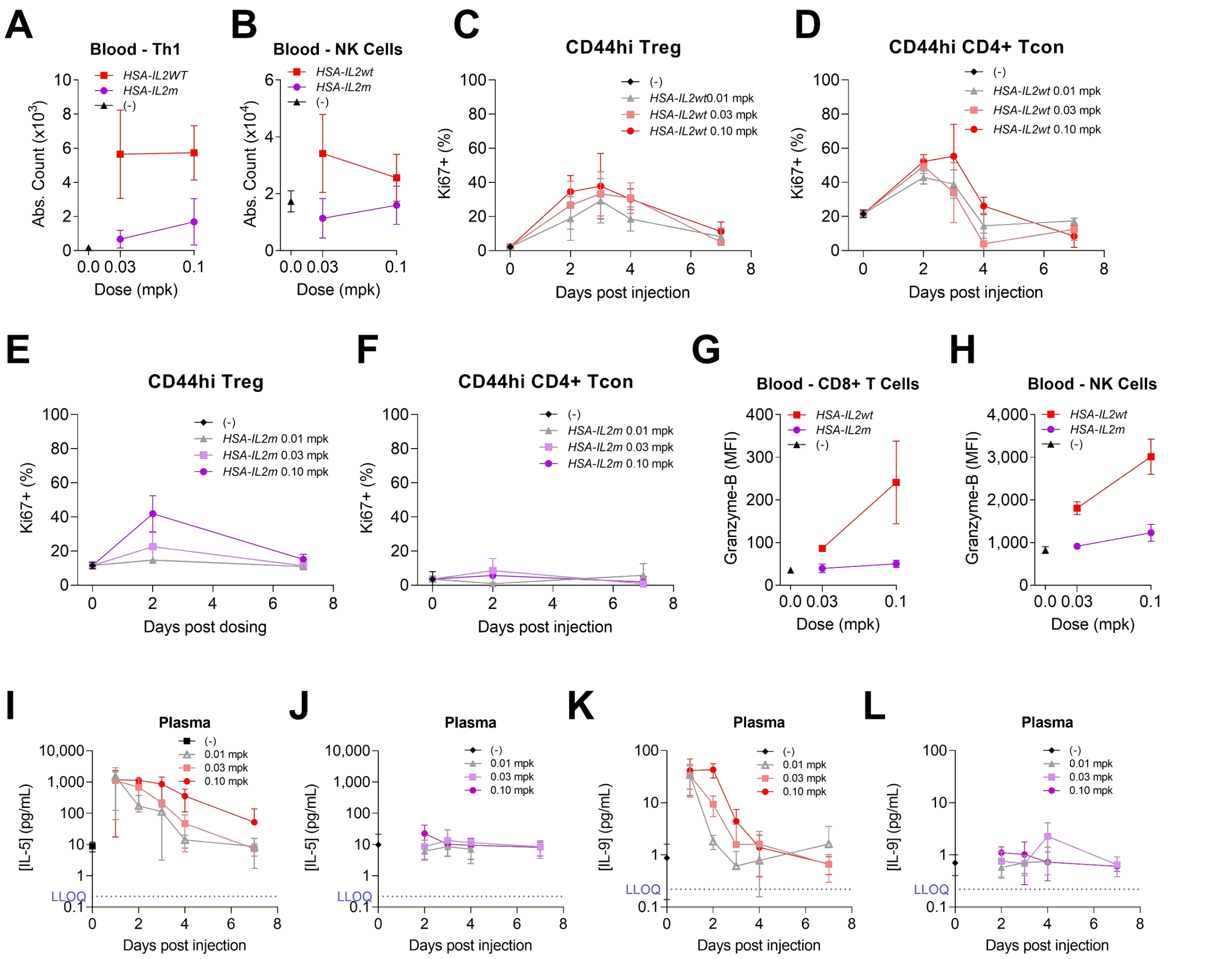

***In vivo* enhanced selectivity of HSA-IL2m towards regulatory T cells in mice**. *HSA-IL2wt* expands proinflammatory A) Th1 cells and B) NK cells in blood (n = 5). Frequency of Ki67 positive C) regulatory T cells and D) conventional CD4 T cells after *HSA-IL2wt* treatment (n = 6). Frequency of Ki67 positive E) regulatory T cells and F) conventional CD4 T cells after *HSA-IL2m* treatment (n=6). *HSA-IL2wt* activates G) CD8 T cells and H) NK cells as shown by upregulation of GZMB (n = 5). Plasma concentration of IL-5 after administration of I) *HSA-IL2wt* or J) *HSA-IL2m* (n = 6). Plasma concentration of IL-9 after administration of K) *HSA-IL2wt* or L) *HSA-IL2m* (n = 6). Data are presented as mean ± s.d.

Supplementary Figure 5:

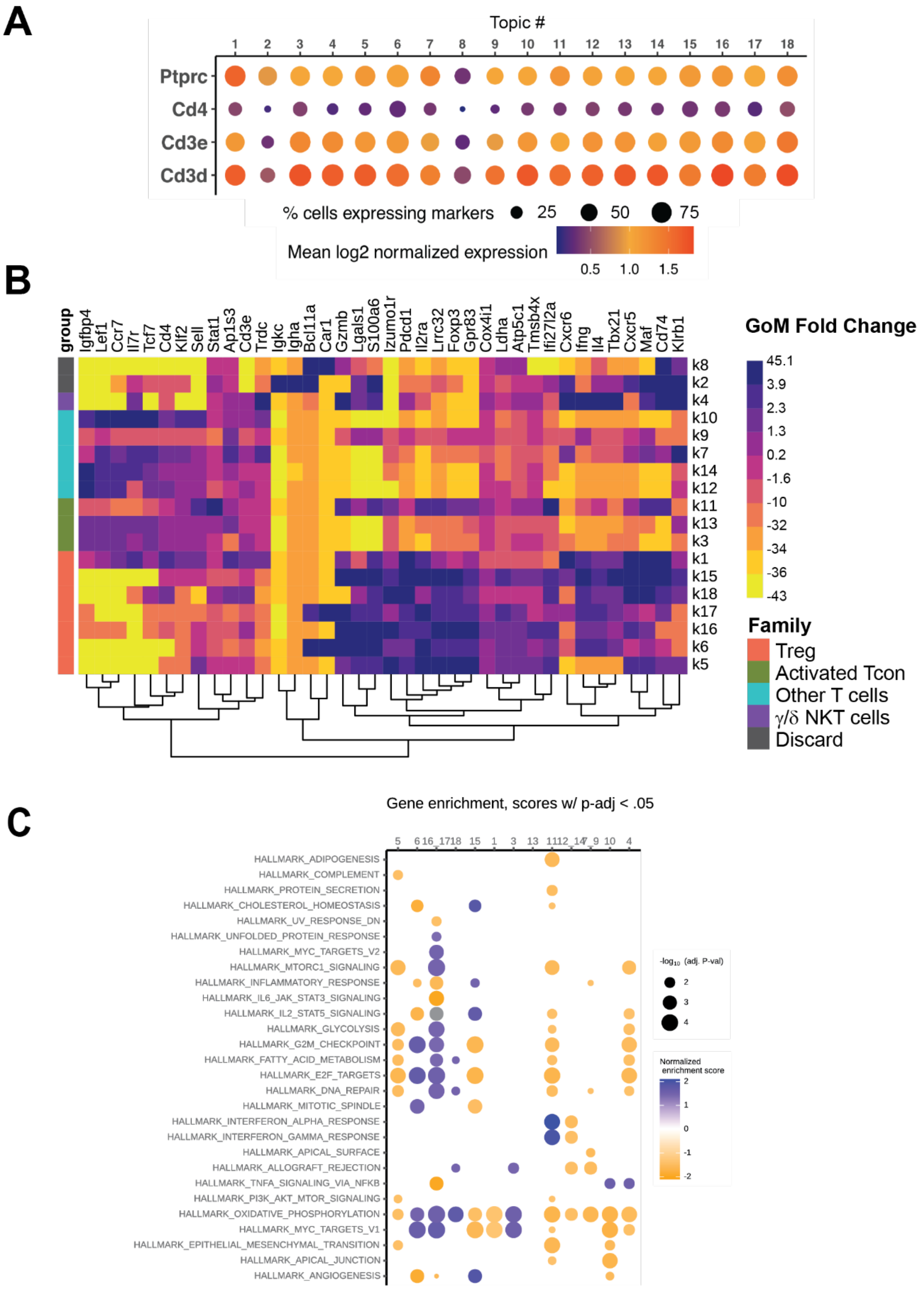

Characterization of CD4 T cells by scRNA-seq. Topic modeling of scRNA-seq data across treatment conditions results in 18 topics. (A) Expression profile of canonical CD4 T cell genes in each topic, according to mean log2 normalization gene expression (color) and the frequency of cells expressing each marker (UMI > 0). (B) Topic-defining genes and CD4 T cell family annotation corresponding to each topic (left bar). Color corresponds to log2-fold-change of the selected topic-defining genes (mean gene expression for all cells assigned to topic k vs all other topics weighted by membership weights). (C) Gene set enrichment analysis using MSigDB Hallmark collection gene sets at false discovery rate < .05. The input to enrichment analysis was based on pairwise cluster comparisons (Welch's *t*-test k). For each topic k, the input log2-fold-change was defined as the minimal log2-fold-change across all pairwise comparisons. Dot color indicates normalized enrichment score. Dot size indicates $-\log_{10}$ P-value adjusted for false discovery rate.

Supplementary Figure 6:

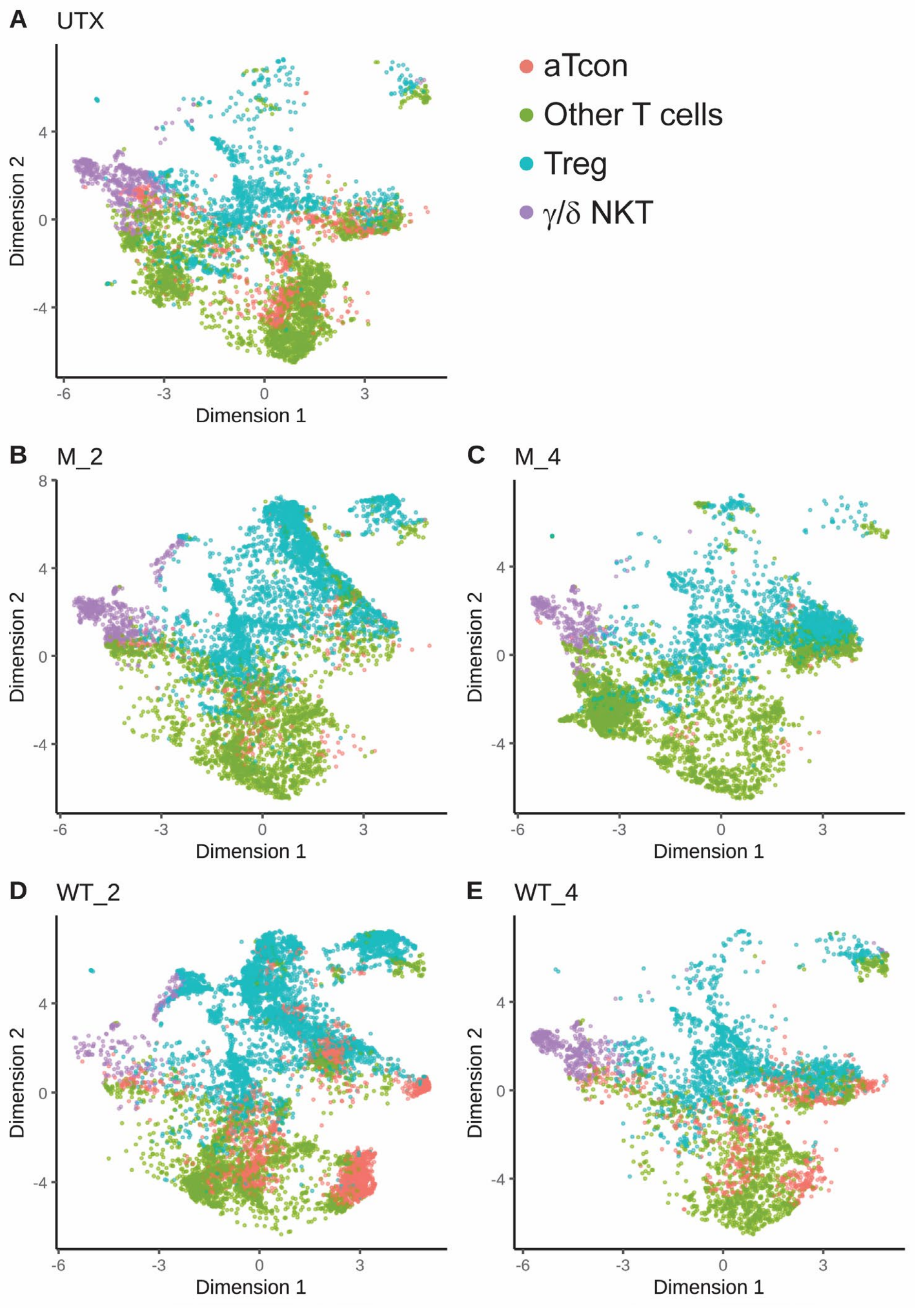

CD4 T cell characterization by treatment condition. UMAP embedding of scRNA-seq profiles (dots) for each treatment condition (A-E), with profiles colored by cell type family (orange: Activated T cells, green: Other T cells, blue: T regulatory cells, purple: g/d NKT cells).

Supplementary Figure 7

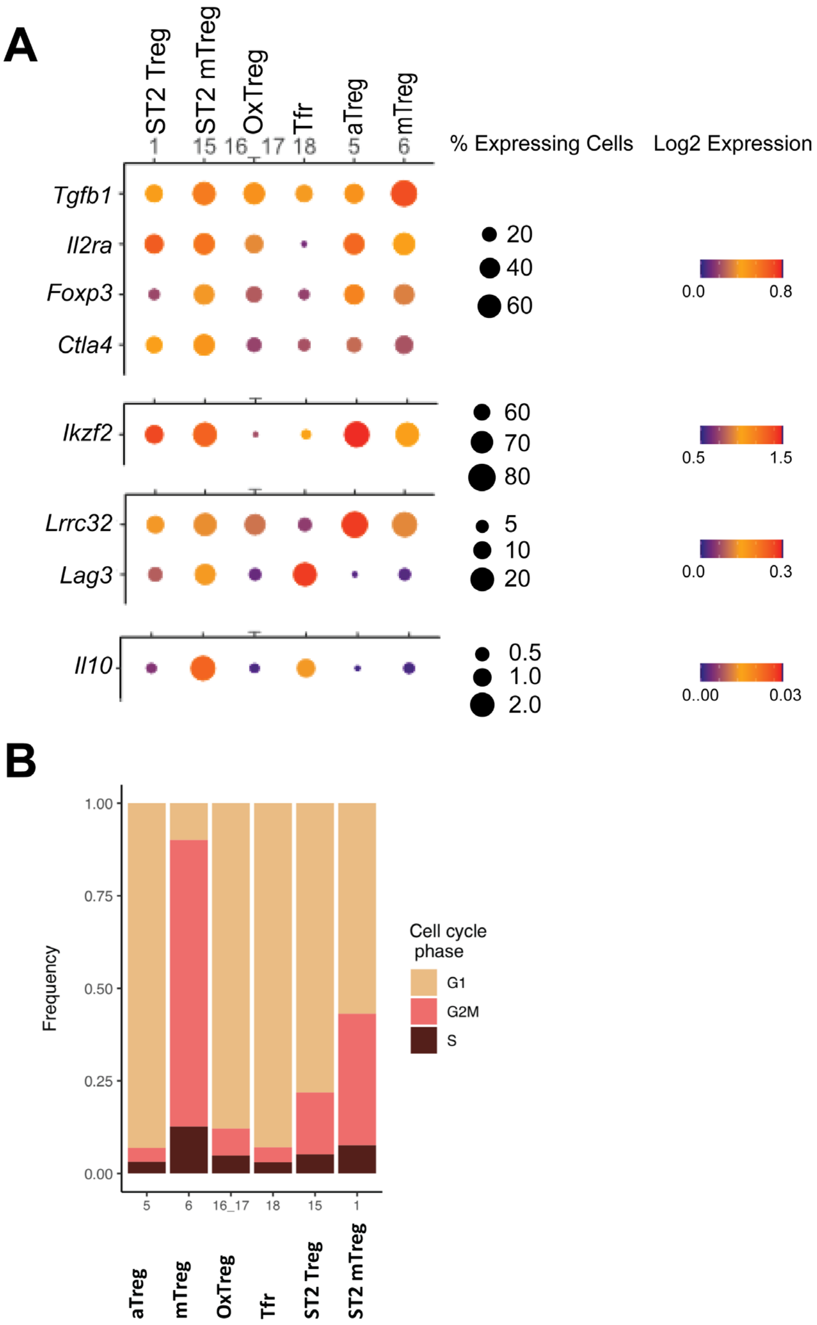

Characterization of regulatory T cells subsets identified by scRNA-seq. (A) Expression profile of canonical Treg genes in each topic, according to mean log2 normalization gene expression (color) and the frequency of cells expressing each marker (UMI > 0). (B) Frequency of cells assigned to a given cell cycle stage by Cyclone.

Supplementary Figure 8

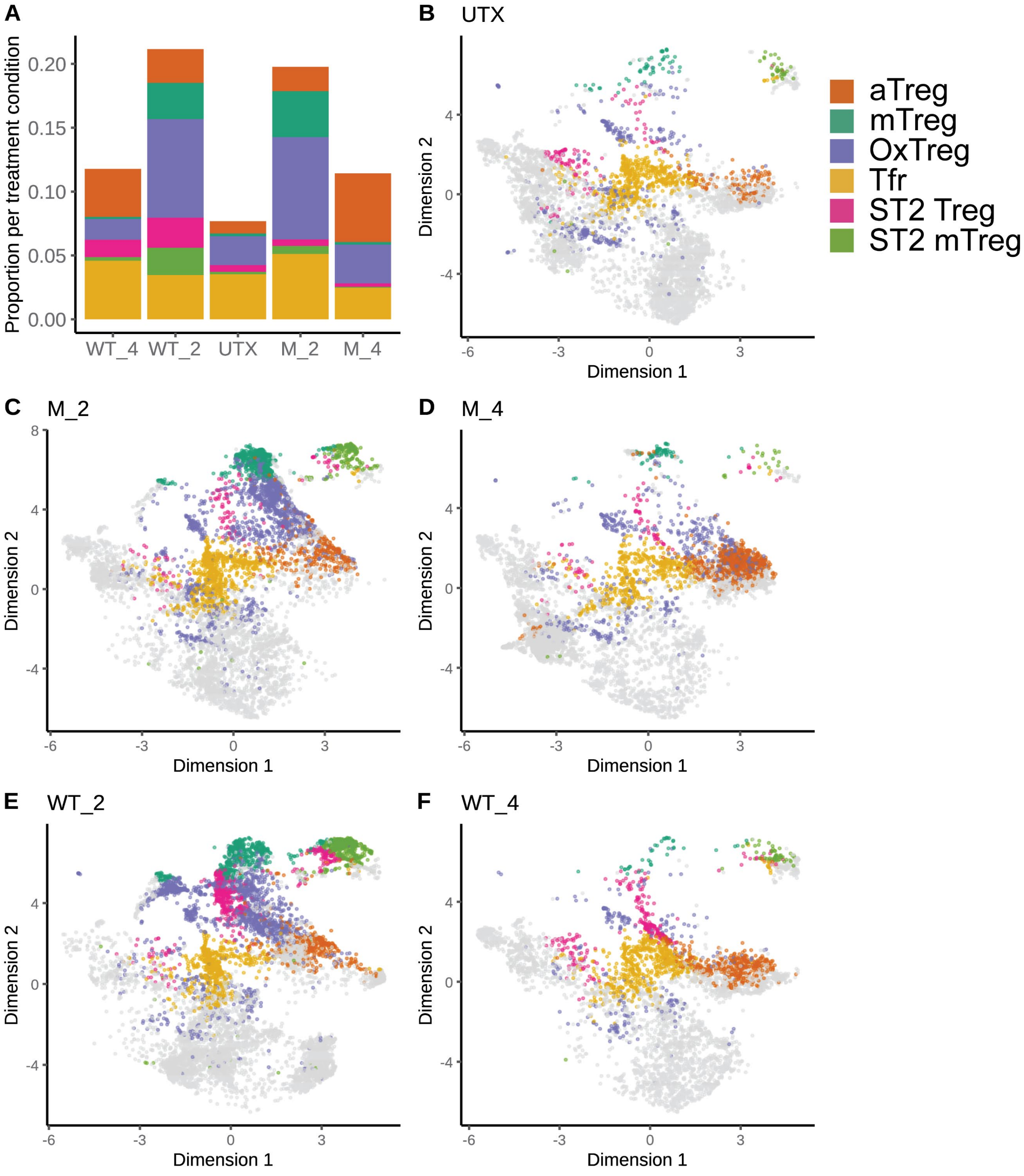

Characterization of Treg topics by treatment condition. (A) Frequency of Treg topics by treatment condition. (B-F) UMAP embedding of scRNA-seq profiles (dots) for each treatment condition, with profiles colored by Treg subtypes.

Supplementary Figure 9

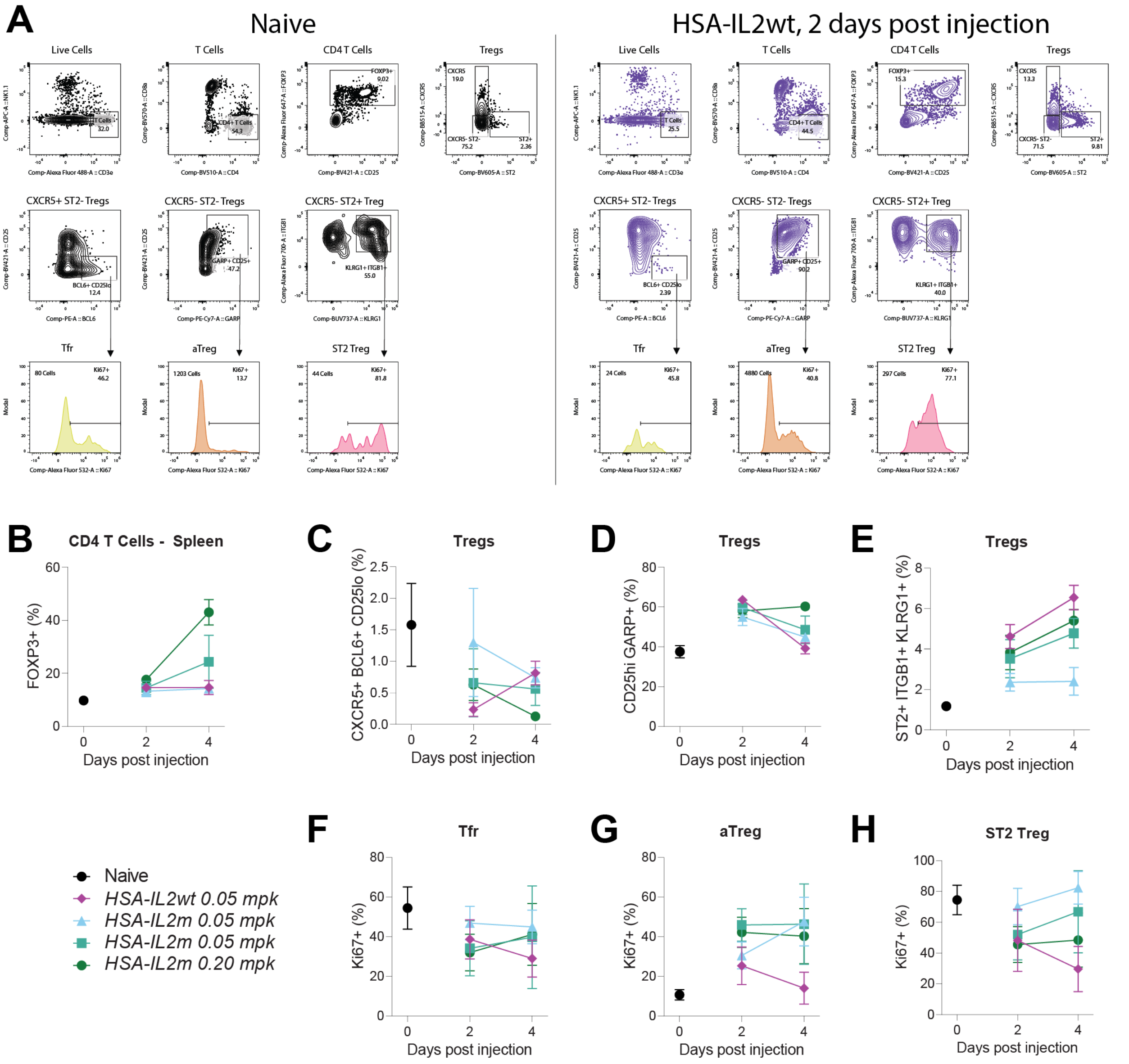

Validation of single cell RNA sequencing by flow cytometry. A) Gating strategy for the identification of Tfr, aTreg and ST2 Treg subsets exemplified on an untreated spleen sample (left) and a *HSA-IL2wt* 0.05 mpk two days post injection spleen sample. B) Quantification of total Tregs (FOXP3+) among CD4 T cells. C) Frequency of CXCR5+ BCL6+ CD25lo (Tfr) cells

among total Tregs. D) Frequency of CD25hi GARP+ (aTreg) cells among total Tregs. E) Frequency of ST2+ ITGB1+ KLRG1+ (ST2 Treg) cells among total Tregs. F) Frequency of Ki67+ cells among Tfr cells. G) Frequency of Ki67+ cells among aTreg cells. H) Frequency of Ki67+ among ST2 Treg cells. For B-H), data is shown as mean +/- standard deviation for n= 5 animals per group.

Cells were stained with biotinylated CXCR5 (1:100) for 15 minutes at 37C. After washing, surface staining was performed for 60 minutes with Fc block (1:100), Live Dead BLUE (1:100), CD3 AF488 (1:400), CD4 BV510 (1:1600), CD8a BV570 (1:400), NK1.1 APC (1:800), CD25 BV421 (1:400), KLRG1 BUV737 (1:400), Streptavidin BB515 (1:200), ST2 BV605 (1:100), ITGB1 AF700 (1:200), Lrrc32/GARP PE/Cy7 (1:100). After fixation cells were incubated overnight with FOXP3 AF647 (1:800), Ki67 AF532 (1:1600), BCL6 PE (1:200). Samples were acquired on a Cytek Aurora flow cytometer.

Supplementary Figure 10

A

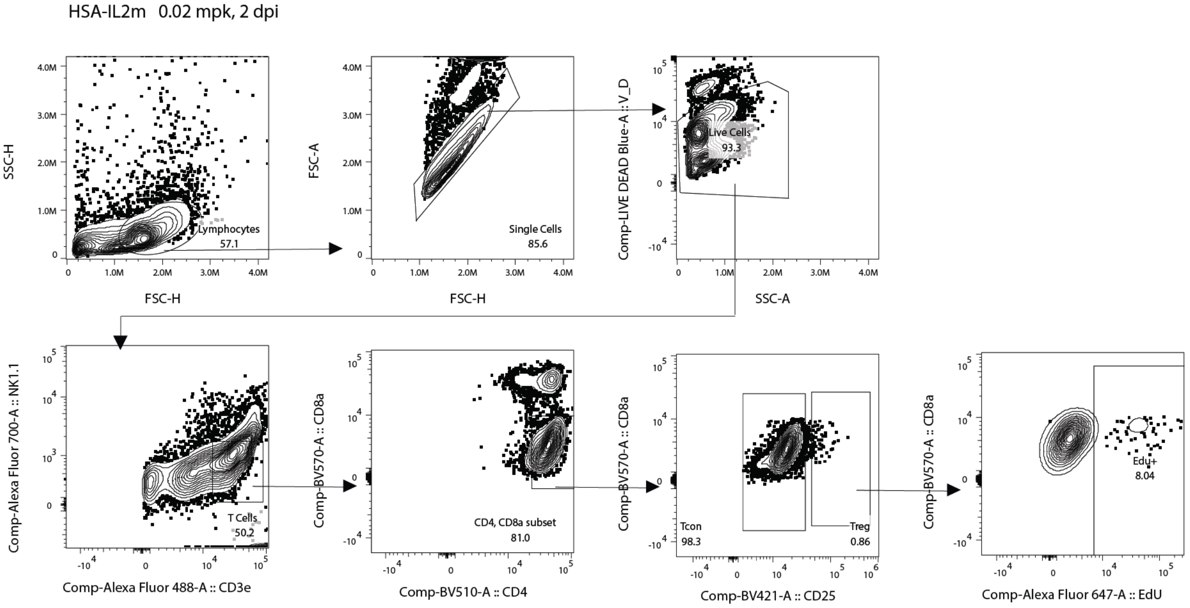

B

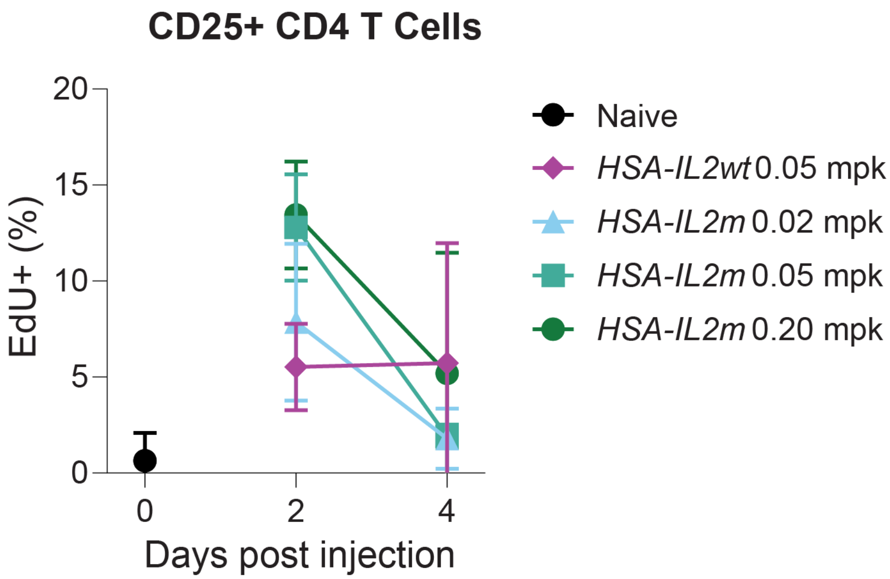

Treg proliferation assay. Splenocytes were incubated 1h *ex vivo* with a thymidine analog compatible for click chemistry (alkyne). Treg cells were stained with viability dye, CD3e, CD4, CD8 and CD25 antibodies and azide Alexa 647. A) Gating strategy is shown on a sample from two days post injection with 0.02 mpk *HSA-IL2m.* B) Quantification of EdU positive CD25+ CD4 T Cells. Data is shown as mean+/- standard deviation for n = 5.

Supplementary Figure 11

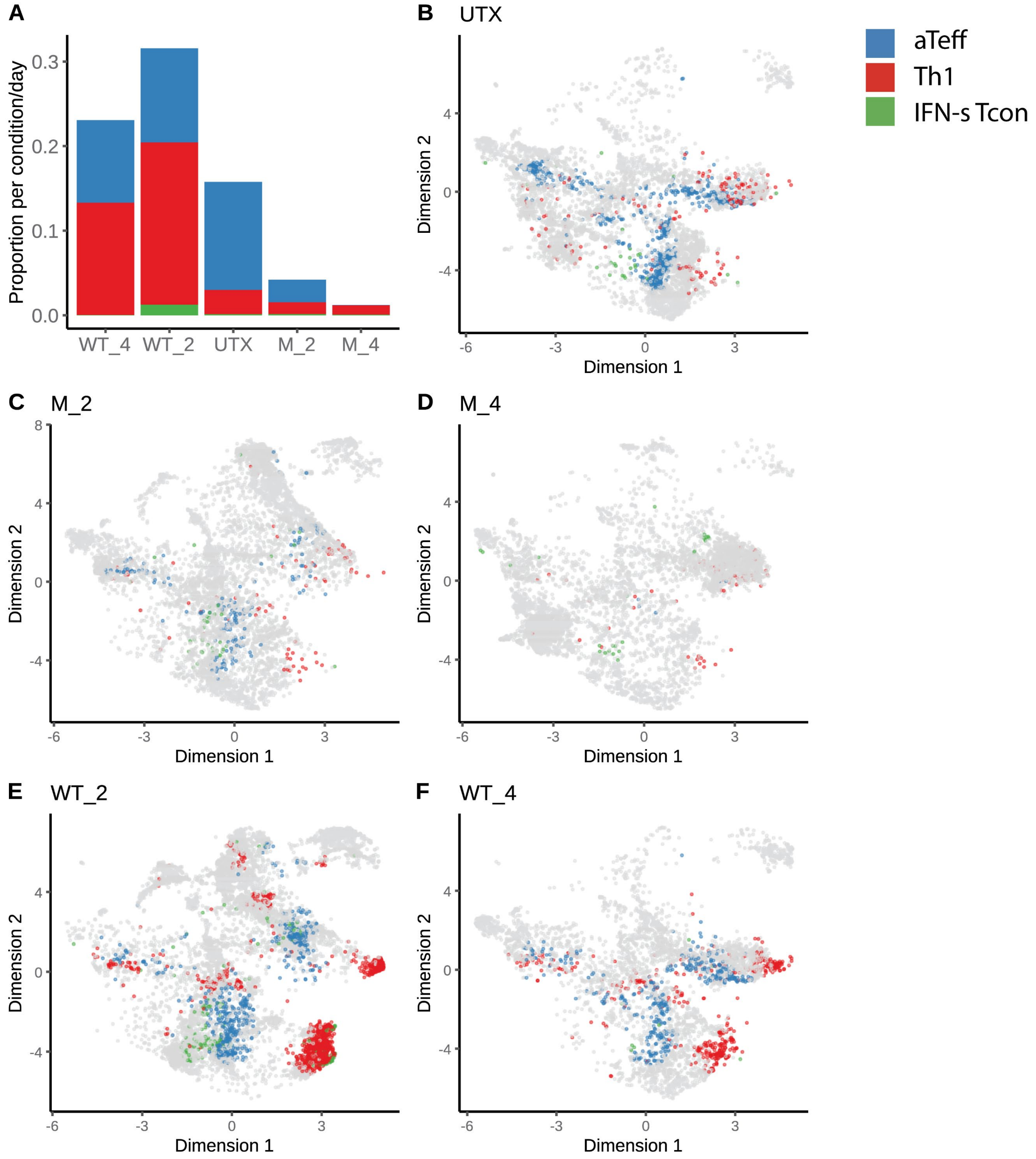

Characterization of activated Tcon topics by treatment condition. (A) Frequency of activated Tcon topics by treatment condition. (B-F) UMAP embedding of scRNA-seq profiles (dots) for each treatment condition, with profiles colored by activated Tcon subtypes.

Supplementary Figure 12

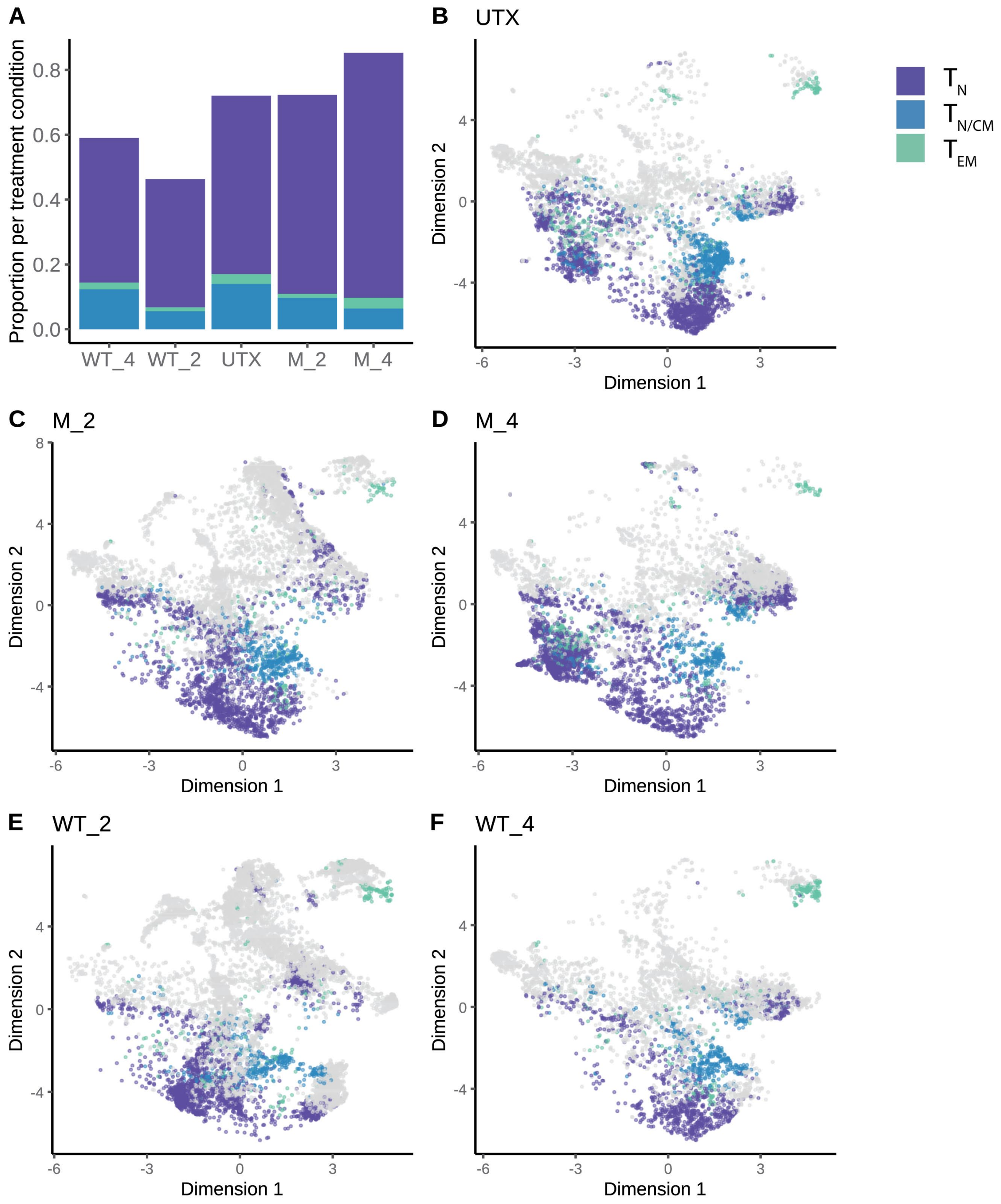

Characterization of other Tcon topics by treatment condition. (A) Frequency of other Tcon topics by treatment condition. (B-F) UMAP embedding of scRNA-seq profiles (dots) for each

treatment condition (B-F), with profiles colored by other Tcon subtypes. Topic 7/9, was characterized by differentially expressed genes involved in cell cycle control (*Cdkn1b*) and fatty acid metabolism (*Acot2*). Given higher *Cd44* and lower *Sell* expression than its counterpart conventional T cell topics, we assigned this topic to effector memory T cells (T_{EM}). Topic 10 was characterized by high expression of *il7r* and *ccr7* which are normally expressed in naïve and central memory T cells, but not in effector memory cells. Furthermore, a transcript associated with quiescence (*Klf3*) was differentially expressed in this subset. Additionally, this topic is enriched for CD4 lineage committing transcription factors (*Satb1, tcf1, lef1)*. We thus labeled this topic as a mix of naïve and central memory T cells ($T_{N/CM}$). Topic 12/14 was the most abundant topic representing ~ 50% of cells. This topic was characterized by transcripts related to cell cycle regulation (*jund, G0s2*), metabolism (*Arl4c*, *Ldlr*, *Igfbp4*) and T cell trafficking regulation (*Pdlim4*). Thus, we assigned this topic to naïve CD4 T cells (T_N).

Supplementary Figure 13

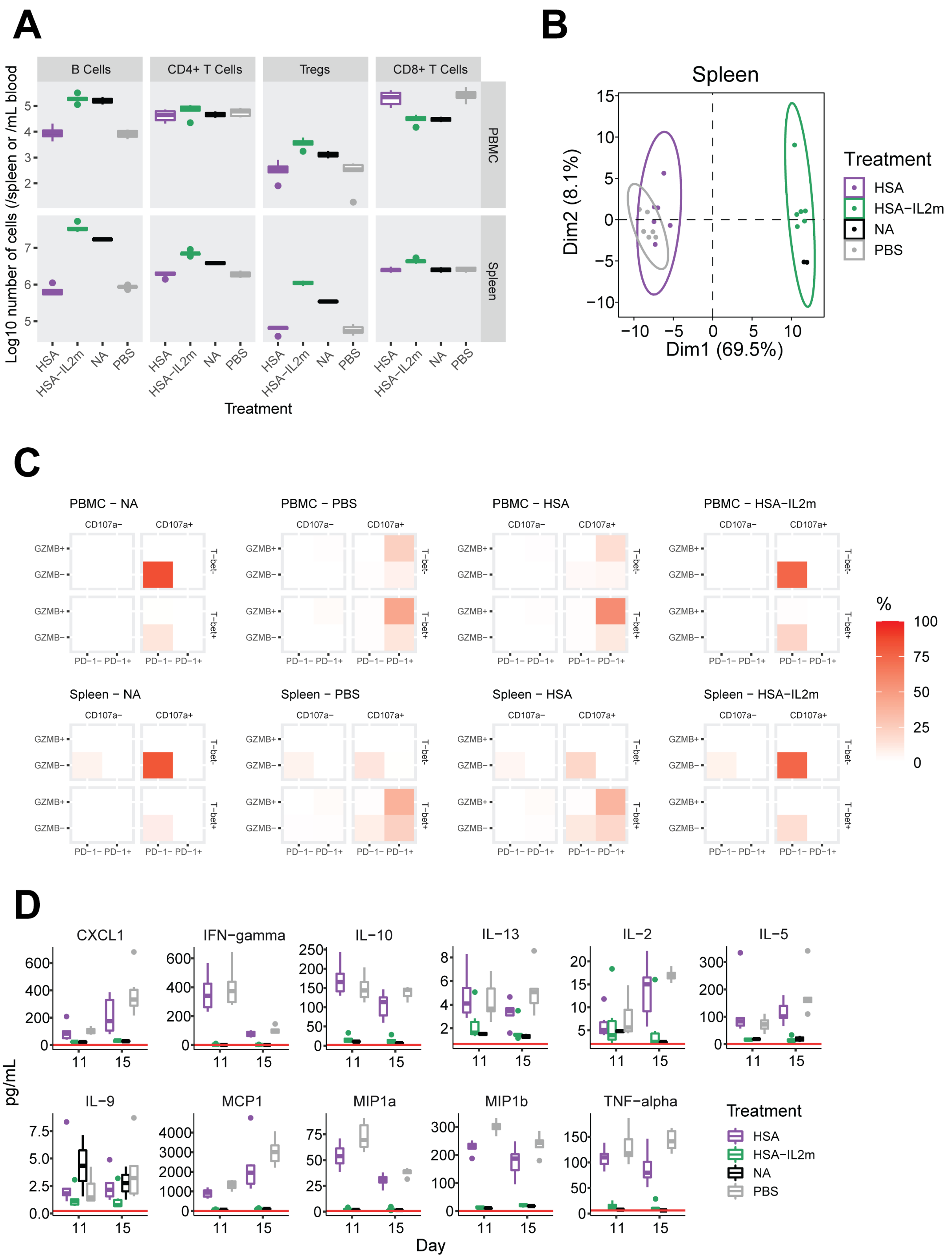

Acute GvHD model. A) Absolute number of B cells, CD4 T cells, CD8 T cells and Tregs in spleen and blood 14 days after cell transfer. B) Principal component projections of animal from blood and splenic flow cytometry data. C) phenotyping of CD8 T cells based on expression of surface PD-1 and intracellular CD107a, GZMB, and T-bet. D) Serum cytokines and chemokines 11 and 15 days after cell transfer (NA: n=2, all other groups: n=6). Box plots center is the median, bounds of box are 1st and 3rd quartile. Upper whisker is the largest value that is no greater than 1.5x the interquartile range plus the third quartile. Lower whisker is the smallest value that is no smaller than the first quartile minus 1.5x the interquartile range

Supplementary Figure 14

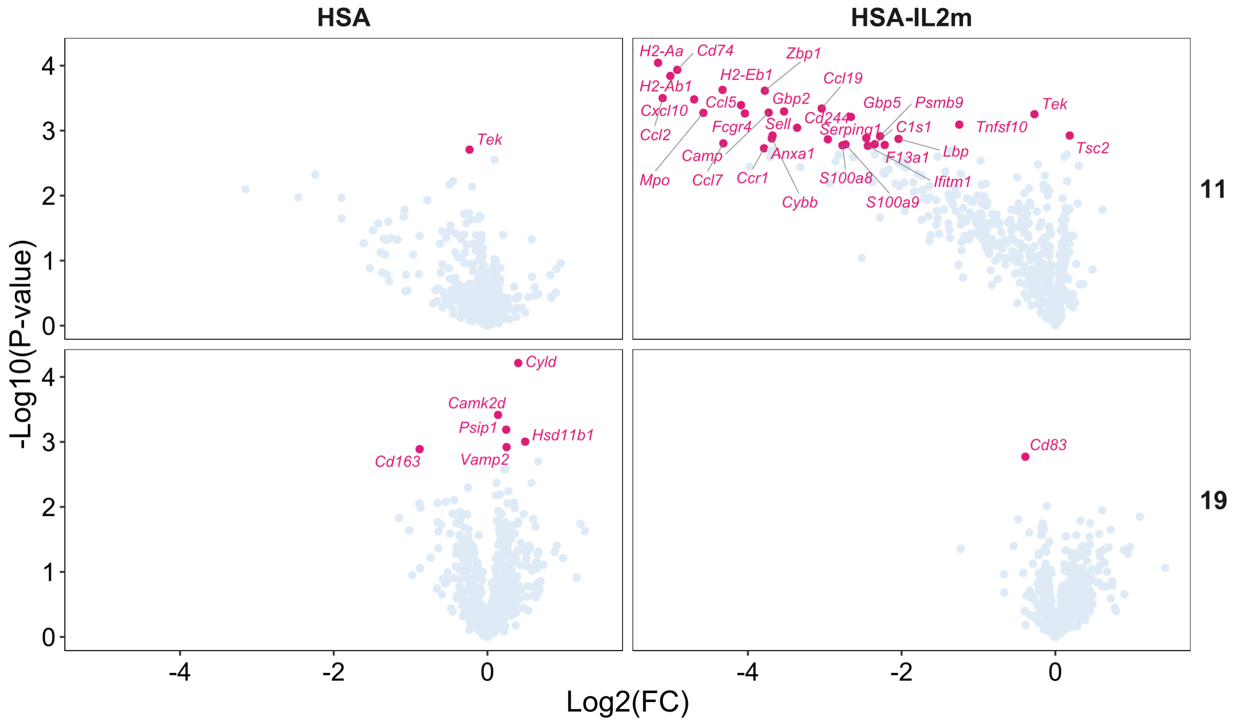

Differential gene expression of spinal cord tissue on day 11 and day 19 (day 1 is the first immunization day) with *HSA* and *HSA-IL2m* treated animals compared to PBS (n = 4). Fold change associated P-values were adjusted using Benjamini-Yekutieli multiple comparison correction.

Supplementary Figure 15

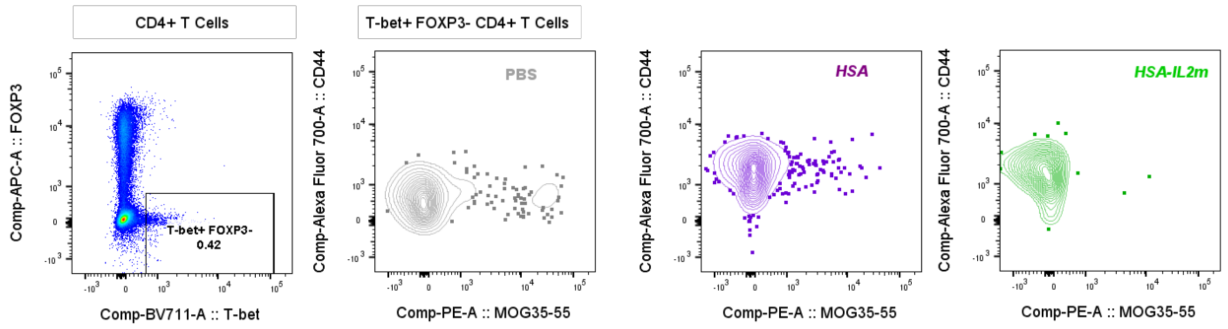

Representative staining for MOG35-55 Tetramer on CD4 T cells in inguinal lymph node samples, 10 days after immunization. CD4 T cells are gated for T-bet+ FOXP3- and then displayed with tetramer staining, shown with a representative sample from each group.

Supplementary Figure 16

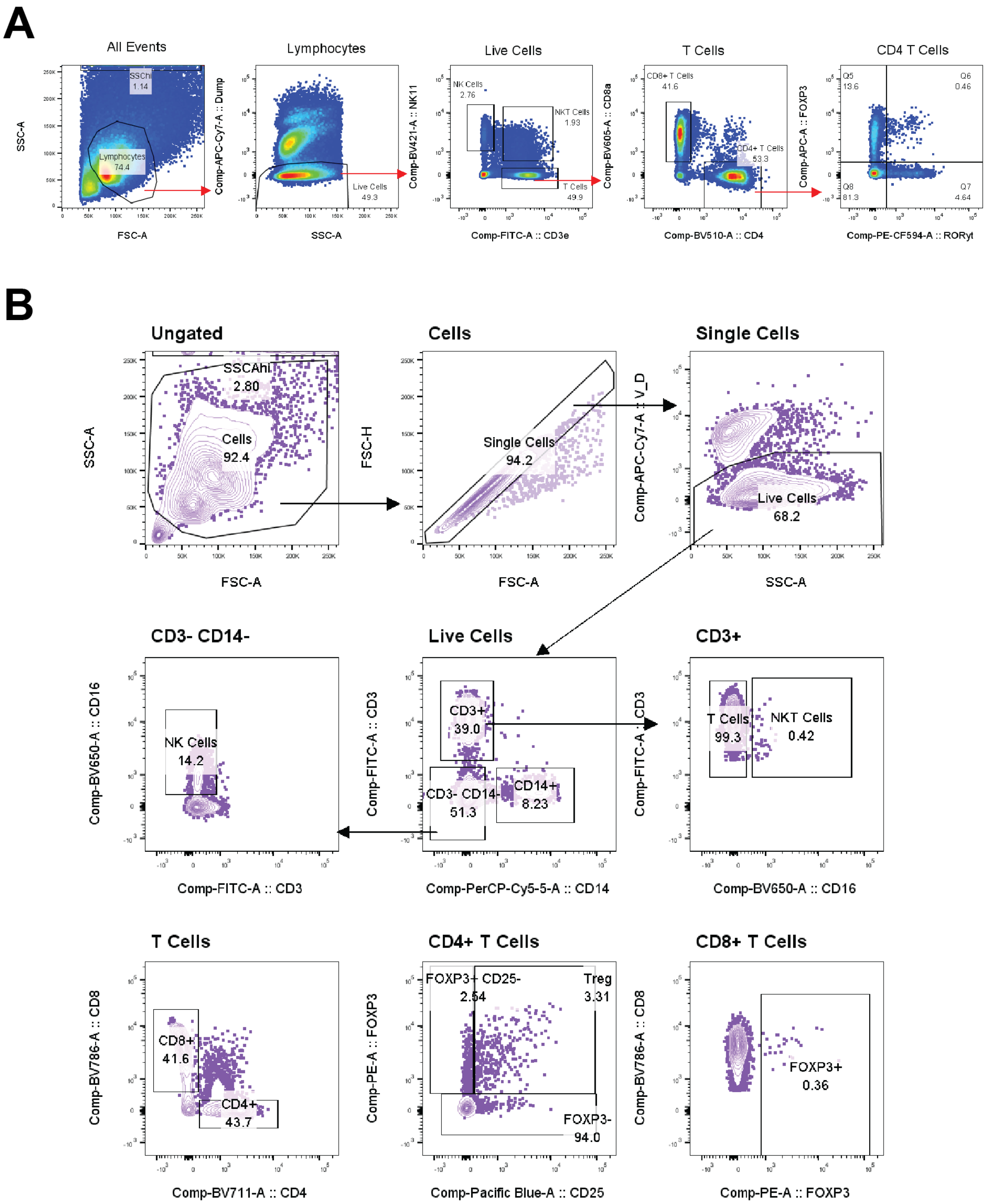

Illustrative flow cytometry gating of murine splenocytes and cynomolgus PBMCs. A) Splenocytes from a representative EAE study. B) Representative sample from untreated cynomolgus monkey PBMC.

2. Supplementary Tables

Supplementary Table 1: Antibody used in the in vitro STAT5 phosphorylation assays

Human PBMC:

Target	Fluor.	Vendor	Catalog #	Clone
CD3	BV510	Biolegend	300448	UCHT1
CD4	BV605	Biolegend	344646	SK3
CD45RA	AF488	Biolegend	304114	HI100
FoxP3	AF647	Biolegend	320014	150D
CD56	PE-Cy7	Biolegend	318318	HCD56
CD8	PerCP-Cy5.5	Biolegend	344710	SK1
pSTAT5	PE	BD	562077	47/Stat5(pY694)

Cyno PBMC

Target	Fluor.	Vendor	Catalog #	Clone
CD4	BV711	Biolegend	317440	OKT4
CD8	BV786	Biolegend	344740	SK1
CD16	BV650	Biolegend	302042	3G8
CD45RA	AF488	BD	555628	5H9
pSTAT5	PE	BD	562077	47/Stat5(pY694)
FOXP3	AF647	Biolegend	320014	150D

Rat splenocytes

Target	Fluor.	Vendor	Catalog #	Clone
CD32	N/A	BD	550271	
CD4	BV711	BD	740723	OX-35
CD8a	PerCP	BD	201712	OX-8
CD161a	BV786	BD	744054	10/78
pSTAT5	PE	BD	562077	47/Stat5(pY694)
FOXP3	AF647	Biolegend	320014	150D

Mouse splenocytes:

Target	Fluor.	Vendor	Catalog #	Clone
Fc Block	N/A	Biolegend	101302	93
CD3e	AF488	Biolegend	100321	145-2C11
CD4	BV786	Biolegend	100453	GK1.5

CD8a	BV421	Biolegend	127643	1A8
CD44	BV510	Biolegend	103044	IM7
NK1.1	BV605	Biolegend	108753	PK136
pSTAT5	PE	BD	562077	47/Stat5(pY694)
FOXP3	AF647	Biolegend	320014	150D

All dilutions at 1:200, Fc block at 1:100.

Supplementary Table 2: Antibodies used for ex vivo flow cytometry studies

Target	Fluorophore	Dilution	Vendor	Catalog #	Clone
Antibodies for mouse cell flow cytometry					
CD103	BV786	1:100	Biolegend	#121439	2E7
CD11b	AF700	1:2000	Biolegend	#100753	53-6.7
CD11b	PerCP/Cy5.5	1:4000	Biolegend	#101228	M1/70
CD19	APC/Cy7	1:2000	Biolegend	#115530	6D5
CD19	PE/Cy7	1:500	Biolegend	#100753	53-6.7
CD25	BV650	1:450	Biolegend	#102038	PC61
CD27	BV421	1:1350	Biolegend	#124223	3A10
CD3	AF488	1:450	Biolegend	#100321	145-2C11
CD3	AF700	1:400	Biolegend	#100216	17A2
CD39	PE-Cy7	1:450	Biolegend	#143806	Duha59
CD4	BV510	1:2000	Biolegend	#100449	GK1.5
CD4	BV605	1:2000	Biolegend	#100451	GK1.5
CD4	BV711	1:2000	Biolegend	#100447	GK1.5
CD4	BV785	1:1350	Biolegend	#100453	GK1.5
CD44	AF488	1:2500	Biolegend	#103016	IM7
CD44	AF700	1:1000	Biolegend	#103026	IM7
CD44	BV510	1:1500	Biolegend	#103044	IM7
CD62L	PE/Cy7	1:4000	Biolegend	#104418	MEL-14
CD73	BV605	1:450	Biolegend	#127215	TY/11.8
CD8a	APC/Cy7	1:2500	Biolegend	#100714	53-6.7
CD8a	BV605	1:2500	Biolegend	#100744	53-6.7
CD8a	PE/594	1:4000	Biolegend	#100762	53-6.7
CD8a	PerCP-Cy5.5	1:1000	Biolegend	#100734	53-6.7
CTLA4	PE-594	1:800	Biolegend	#106318	UC10-4B9
CXCR5	Biotin	1:20	Biolegend	#145504	L138D7
FoxP3	AF647	1:200	Biolegend	#320014	150D
FoxP3	PE	1:800	Biolegend	#126404	MF-14
GITR	AF488	1:2500	Biolegend	#120211	YGITR765
GR-B	AF647	1:320	Biolegend	#515406	GB11
H2-Kb	BV421	1:900	Biolegend	#116513	AF6-88.5
H2-Kd	PE	1:250	eBiosciences	#17-5957-82	SF1-1.1.1
IA/IE	AF700	1:2000	Biolegend	#107622	M5/114.15.2
IFNy	BV785	1:800	Biolegend	#505838	XMG1.2
IL-10	PE-594	1:2000	Biolegend	#505034	JES5-16E3
IL-17A	BV650	1:4050	Biolegend	#506930	TC11-18H10.1
Ki67	PerCP/Cy5.5	1:800	Biolegend	#652424	16A8

MOG35-55 Tetramer	PE	1:5	MBL International	#TS-M704-1	-
NK1.1	APC/Cy7	1:900	Biolegend	#108724	PK136
NK1.1	BV421	1:900	Biolegend	#108741	PK136
NK1.1	BV605	1:450	Biolegend	#108753	PK136
NKp46	FITC	1:300	Biolegend	#137606	29A1.4
PD-1	BV786	1:200	Biolegend	#135225	29F.1A12
PD-1	BV711	1:1350	Biolegend	#135231	29F.1A12
RORyt	PE-594	1:800	BD	#562684	Q31-378
Streptavidin	PE/Cy7	1:500	Biolegend	#	N/A
Tbet	BV711	1:800	Biolegend	#644820	4B10
TIGIT	BV421	1:400	Biolegend	#142111	1G9
**Antibodies for cynomolgus monkey studies**					
CD14	PerCP/Cy5.5	1:200	Biolegend	#301824	M5E2
CD16	BV650	1:400	Biolegend	#302042	3G8
CD20	PE/Cy7	1:5000	Biolegend	#302312	2H7
CD25	BV421	1:50	Biolegend	#356114	M-A251
CD27	BV510	1:800	Biolegend	#302836	O323
CD3	AF488	1:100	BD	#557705	SP34-2
CD3	BV605	1:100	BD	#562994	SP34-2
CD39	BV510	1:400	Biolegend	# 328219	A1
CD4	BV711	1:400	Biolegend	#317440	OKT4
CD45RA	APC	1:3200	BD	#561210	5H9
CD45RA	FITC	1:1600	BD	#555626	5H9
CD45RO	AF700	1:50	Biolegend	#304218	UCHL1
CD69	BV605	1:100	Biolegend	#310938	FN50
CD8	BV786	1:100	Biolegend	#344740	SK1
CTLA4	PE/594	1:400	Biolegend	# 369616	BNI3
FoxP3	PE	1:400	Biolegend	#320108	206D
FoxP3	AF647	1:200	Biolegend	#320014	150D
GITR	APC	1:50	Biolegend	# 311610	621
GZMB	PerCP/Cy5.5	1:800	Biolegend	#372212	QA16A02
HLA-DR	BV510	1:150	Biolegend	#307646	L243
Ki67	PE/Cy7	1:1600	Biolegend	# 350526	Ki-67
PD-1	PE/Cy7	1:400	Biolegend	#329918	EH12.2H7
T-bet	PE/594	1:3200	Biolegend	#644828	4B10
