# Supplementary material for: Selective activation and expansion of regulatory T cells using lipid encapsulated mRNA encoding a long-acting IL-2 mutein
Source: Nat Commun. 2022 Jul 5;13:3866. doi: 10.1038/s41467-022-31130-9 (PMC9256694; doi:10.1038/s41467-022-31130-9)
Supplement: Supplementary file 2 — Reporting Summary [file 41467_2022_31130_MOESM2_ESM.pdf]

nature research

Corresponding author(s): Eric Huang, Seymour de Picciotto

Last updated by author(s): Mar 30, 2022

Reporting Summary

Nature Research wishes to improve the reproducibility of the work that we publish. This form provides structure for consistency and transparency in reporting. For further information on Nature Research policies, see our Editorial Policies and the Editorial Policy Checklist.

Statistics

For all statistical analyses, confirm that the following items are present in the figure legend, table legend, main text, or Methods section.

n/a	Confirmed	
☐	☒	The exact sample size (*n*) for each experimental group/condition, given as a discrete number and unit of measurement
☐	☒	A statement on whether measurements were taken from distinct samples or whether the same sample was measured repeatedly
☐	☒	The statistical test(s) used AND whether they are one- or two-sided<br>*Only common tests should be described solely by name; describe more complex techniques in the Methods section.*
☐	☒	A description of all covariates tested
☐	☒	A description of any assumptions or corrections, such as tests of normality and adjustment for multiple comparisons
☐	☒	A full description of the statistical parameters including central tendency (e.g. means) or other basic estimates (e.g. regression coefficient) AND variation (e.g. standard deviation) or associated estimates of uncertainty (e.g. confidence intervals)
☐	☒	For null hypothesis testing, the test statistic (e.g. *F*, *t*, *r*) with confidence intervals, effect sizes, degrees of freedom and *P* value noted<br>*Give P values as exact values whenever suitable.*
☒	☐	For Bayesian analysis, information on the choice of priors and Markov chain Monte Carlo settings
☒	☐	For hierarchical and complex designs, identification of the appropriate level for tests and full reporting of outcomes
☒	☐	Estimates of effect sizes (e.g. Cohen's *d*, Pearson's *r*), indicating how they were calculated

Our web collection on statistics for biologists contains articles on many of the points above.

Software and code

Policy information about availability of computer code

Data collection	Flow cytometry data was collected using BD FACS Diva and SpectroFlo
Data analysis	R/Bioconductor softwares: R v4.1.2, Bioconductor v3.13, BiocManager v1.30.16, DropletUtils v1.14.2, edgeR v3.34.1, fastTopics v0.5-25, fgsea v3.12, limma v3.48.3, msigdbr v7.2.1, scran v1.20.1, scuttle v1.2.1, SingleCellExperiment v1.14.1, SummarizedExperiment v1.22.0; Python: python v2.7.2, scanpy v1.7.2; Other algorithms: bustools v0.39.3. Flow cytometry: Flowjo version 10

For manuscripts utilizing custom algorithms or software that are central to the research but not yet described in published literature, software must be made available to editors and reviewers. We strongly encourage code deposition in a community repository (e.g. GitHub). See the Nature Research guidelines for submitting code & software for further information.

Data

Policy information about availability of data

All manuscripts must include a data availability statement. This statement should provide the following information, where applicable:

- Accession codes, unique identifiers, or web links for publicly available datasets
- A list of figures that have associated raw data
- A description of any restrictions on data availability

The code and processed data associated with single-cell RNA-seq data analysis are available at GitHub: https://github.com/modernatx/selective_activation_expansion_Treg_using_lipid_encapsulated_mRNA_encoding_longacting_IL2_mutein.

Field-specific reporting

Please select the one below that is the best fit for your research. If you are not sure, read the appropriate sections before making your selection.

- [x] Life sciences
- [ ] Behavioural & social sciences
- [ ] Ecological, evolutionary & environmental sciences

For a reference copy of the document with all sections, see nature.com/documents/nr-reporting-summary-flat.pdf

Life sciences study design

All studies must disclose on these points even when the disclosure is negative.

Sample size: Sample size was determined from power analysis using available data whenever possible.

Data exclusions: For scRNASeq, EmptyDrops was used to distinguish droplets containing cells and droplets containing cell-free RNA (FDR threshold < .01, niter=100,000 for the Monte Carlo p-value calculation). Cells were further excluded if they were associated with low number of molecules (< 500 total molecules) or low number of genes detected (< 500 genes). Low count genes were excluded to retain genes detected in > 0.01% of the cells

Replication: Main findings of scRNAseq were confirmed using other methods twice and were successful. All non-scRNAseq studies were repeated at least twice and the replication were succesful.

Randomization: For the non human primate studies, animals were randomized into group while maintaining similar average body weight across groups. For all other studies, allocation was random.

Blinding: The investigator in the EAE studies was blinded to the nature of the test articles. For all studies, the test articles were blinded to the animal facility personnel. During acquisition and data analysis, investigators were not blinded as no bias could be introduced.

Reporting for specific materials, systems and methods

We require information from authors about some types of materials, experimental systems and methods used in many studies. Here, indicate whether each material, system or method listed is relevant to your study. If you are not sure if a list item applies to your research, read the appropriate section before selecting a response.

Materials & experimental systems

n/a	Involved in the study	
	x	Antibodies
	x	Eukaryotic cell lines
x		Palaeontology and archaeology
	x	Animals and other organisms
x		Human research participants
x		Clinical data
x		Dual use research of concern

Methods

n/a	Involved in the study	
x		ChIP-seq
	x	Flow cytometry
x		MRI-based neuroimaging

Antibodies

Antibodies used:

Antibodies for mouse cell flow cytometry:
Fc Block, Dilution 1:100, Biolegend #101302, Clone 93
BCL6 PE, Dilution 1:200, BD Biosciences, Catalog 561522, Clone K112-91
CD11b AF700, Dilution 1:2000, Biolegend, Catalog #100753, Clone 53-6.7;
CD11b PerCP/Cy5.5, Dilution 1:4000, Biolegend, Catalog #101228, Clone M1/70;
CD19 APC/Cy7, Dilution 1:2000, Biolegend, Catalog #115530, Clone 6D5;
CD19 PE/Cy7, Dilution 1:500, Biolegend, Catalog #100753, Clone 53-6.7;
CD25 BV650, Dilution 1:450, Biolegend, Catalog #102038, Clone PC61;
CD27 BV421, Dilution 1:1350, Biolegend, Catalog #124223, Clone LG 3A10;
CD3 AF488, Dilution 1:450, Biolegend, Catalog #100321, Clone 145-2C11;
CD3 AF700, Dilution 1:400, Biolegend, Catalog #100216, Clone 17A2;
CD39 PE-Cy7, Dilution 1:450, Biolegend, Catalog #143806, Clone Duha59;
CD4 BV510, Dilution 1:2000, Biolegend, Catalog #100449, Clone GK1.5;
CD4 BV605, Dilution 1:2000, Biolegend, Catalog #100451, Clone GK1.5;
CD4 BV711, Dilution 1:2000, Biolegend, Catalog #100447, Clone GK1.5;
CD4 BV785, Dilution 1:1350, Biolegend, Catalog #100453, Clone GK1.5;
CD44 AF488, Dilution 1:2500, Biolegend, Catalog #103016, Clone IM7;
CD44 AF700, Dilution 1:1000, Biolegend, Catalog #103026, Clone IM7;

CD44 BV510, Dilution 1:1500, Biolegend, Catalog #103044, Clone IM7;
CD62L PE/Cy7, Dilution 1:4000, Biolegend, Catalog #104418, Clone MEL-14;
CD73 BV605, Dilution 1:450, Biolegend, Catalog #127215, Clone TY/11.8;
CD8a APC/Cy7, Dilution 1:2500, Biolegend, Catalog #100714, Clone 53-6.7;
CD8a BV605, Dilution 1:2500, Biolegend, Catalog #100744, Clone 53-6.7;
CD8a PE/594, Dilution 1:4000, Biolegend, Catalog #100762, Clone 53-6.7;
CD8a PerCP-Cy5.5, Dilution 1:1000, Biolegend, Catalog #100734, Clone 53-6.7;
CD8a BV570, Dilution 1:400, Biolegend, Catalog #100740, Clone 53-6.7
CTLA4 PE-594, Dilution 1:800, Biolegend, Catalog #106318, Clone UC10-4B9;
CXCR5 Biotin, Dilution 1:20, Biolegend, Catalog #145504, Clone L138D7;
FoxP3 AF647, Dilution 1:200, Biolegend, Catalog #320014, Clone 150D;
FoxP3 PE, Dilution 1:800, Biolegend, Catalog #126404, Clone MF-14;
GITR AF488, Dilution 1:2500, Biolegend, Catalog #120211, Clone YGITR765;
GR-B AF647, Dilution 1:320, Biolegend, Catalog #515406, Clone GB11;
H2-Kb BV421, Dilution 1:900, Biolegend, Catalog #116513, Clone AF6-88.5;
H2-Kd PE, Dilution 1:250, eBiosciences, Catalog #17-5957-82, Clone SF1-1.1.1;
IA/IE AF700, Dilution 1:2000, Biolegend, Catalog #107622, Clone M5/114.15.2;
IFNy*4 BV785, Dilution 1:800, Biolegend, Catalog #505838, Clone XMG1.2;
IL-10*3 PE-594, Dilution 1:2000, Biolegend, Catalog #505034, Clone JES5-16E3;
IL-17A*2 BV650, Dilution 1:4050, Biolegend, Catalog #506930, Clone TC11-18H10.1;
ITGB1 AF700, Dilution 1:200, Biolegend, Catalog #102218, Clone HMB1.1
Ki67 PerCP/Cy5.5, Dilution 1:800, Biolegend, Catalog #652424, Clone 16A8;
Ki67 AF532, Dilution 1:1600, ThermoFisher, Catalog #58-5698-82, Clone SolA15;
KLRG1 BUV737, Dilution 1:400, BD Biosciences, Catalog #741812, Clone 2F1
Lrrc32 PE/Cy7, Dilution 1:100, Biolegend, Catalog #142910, Clone F011.5
MOG35-55 Tetramer PE, Dilution 1:5, MBL International, Catalog #TS-M704-1;
NK1.1 APC, Dilution 1:800, Biolegend, Catalog #108710, Clone PK136
NK1.1 APC/Cy7, Dilution 1:900, Biolegend, Catalog #108724, Clone PK136;
NK1.1 BV421, Dilution 1:900, Biolegend, Catalog #108741, Clone PK136;
NK1.1 BV605, Dilution 1:450, Biolegend, Catalog #108753, Clone PK136;
NKp46 FITC, Dilution 1:300, Biolegend, Catalog #137606, Clone 29A1.4;
PD-1 BUV395, Dilution 1:100, BD Biosciences, Catalog #565102, Clone TY25
PD-1 BV786, Dilution 1:200, Biolegend, Catalog #135225, Clone 29F.1A12;
PD-1 BV711, Dilution 1:1350, Biolegend, Catalog #135231, Clone 29F.1A12;
RORγt PE-594, Dilution 1:800, BD, Catalog #562684, Clone Q31-378;
Streptavidin PE/Cy7, Dilution 1:500, Biolegend, Catalog #, Clone N/A;
Streptavidin BB515, Dilution 1:500, BD Biosciences, Catalog #564453, Clone N/A;
ST2 BV605, Dilution 1:100, Biolegend, Catalog #145323, Clone DIH9;
Tbet BV711, Dilution 1:800, Biolegend, Catalog #644820, Clone 4B10;
TIGIT BV421, Dilution 1:400, Biolegend, Catalog #142111, Clone 1G9;

Antibodies for cynomolgus monkey studies:
CD14 PerCP/Cy5.5, Dilution 1:200, Biolegend, Catalog #301824, Clone M5E2;
CD16 BV650, Dilution 1:400, Biolegend, Catalog #302042, Clone 3G8;
CD20 PE/Cy7, Dilution 1:5000, Biolegend, Catalog #302312, Clone 2H7;
CD25 BV421, Dilution 1:50, Biolegend, Catalog #356114, Clone M-A251;
CD27 BV510, Dilution 1:800, Biolegend, Catalog #302836, Clone O323;
CD3 AF488, Dilution 1:100, BD, Catalog #557705, Clone SP34-2;
CD3 BV605, Dilution 1:100, BD, Catalog #562994, Clone SP34-2;
CD39 BV510, Dilution 1:400, Biolegend, Catalog # 328219, Clone A1;
CD4 BV711, Dilution 1:400, Biolegend, Catalog #317440, Clone OKT4;
CD45RA APC, Dilution 1:3200, BD, Catalog #561210, Clone 5H9;
CD45RA FITC, Dilution 1:1600, BD, Catalog #555626, Clone 5H9;
CD45RO AF700, Dilution 1:50, Biolegend, Catalog #304218, Clone UCHL1;
CD69 BV605, Dilution 1:100, Biolegend, Catalog #310938, Clone FN50;
CD8 BV786, Dilution 1:100, Biolegend, Catalog #344740, Clone SK1;
CTLA4 PE/594, Dilution 1:400, Biolegend, Catalog # 369616, Clone BNI3;
FoxP3 PE, Dilution 1:400, Biolegend, Catalog #320108, Clone 206D;
FoxP3 AF647, Dilution 1:200, Biolegend, Catalog #320014, Clone 150D;
GITR APC, Dilution 1:50, Biolegend, Catalog # 311610, Clone 621;
GZMB PerCP/Cy5.5, Dilution 1:800, Biolegend, Catalog #372212, Clone QA16A02;
HLA-DR BV510, Dilution 1:150, Biolegend, Catalog #307646, Clone L243;
Ki67 PE/Cy7, Dilution 1:1600, Biolegend, Catalog # 350526, Clone Ki-67;
PD-1 PE/Cy7, Dilution 1:400, Biolegend, Catalog #329918, Clone EH12.2H7;
T-bet PE/594, Dilution 1:3200, Biolegend, Catalog #644828, Clone 4B10;

Human cells:
CD3 BV510 Biolegend 300448 UCHT1, 1:200

	CD4 BV605 Biolegend 344646 SK3, 1:200<br>CD45RA AF488 Biolegend 304114 HI100, 1:200<br>FoxP3 AF647 Biolegend 320014 150D, 1:200<br>CD56 PE-Cy7 Biolegend 318318 HCD56, 1:200<br>CD8 PerCP-Cy5.5 Biolegend 344710 SK1, 1:200<br>pSTAT5 PE BD 562077 47/Stat5(pY694), 1:20<br>Rat cells:<br>CD32 N/A BD 550271<br>CD4 BV711 BD 740723 OX-35, 1:200<br>CD8a PerCP BD 201712 OX-8, 1:200<br>CD161a BV786 BD 744054 10/78, 1:200<br>pSTAT5 PE BD 562077 47/Stat5(pY694), 1:20<br>FOXP3 AF647 Biolegend 320014 150D, 1:200
Validation	Validation of commercial antibodies was done by regular quality control for each lot by the manufacturer (e.g. Biolegend)<br><br>The specificity and sensitivity of each antibody is thoroughly validated in the New Product Development stage. This is done by staining multiple target cells with either single- or multi-color analysis or by other testing approaches. The QC specifications and testing SOPs and gold standard for each product are then developed.<br><br>The functional performance of each batch of BioLegend products is strictly QC-tested according to the established QC procedures. In general, each product is tested using the following criteria:<br><br>Staining of 1-3 target cell types with either single- or multi-color analysis detailed in the QC specification (including positive and negative controls). The tested cells can be primary cells and/or cell lines known to be positive or negative for the target antigen.<br>Each batch product is validated by QC testing with a series of dilutions to make sure the product is working within expected antibody titer range.<br><br>Each batch is compared to an internally established "gold standard" to maintain batch-to-batch consistency.<br>When applicable, our products are side-by-side tested with our competitors' products to make sure that BioLegend's products exceed or are at least the same quality.<br>For most tandem dye-conjugated products, color compensation is examined in order to verify tandem integrity.

Eukaryotic cell lines

Policy information about cell lines

Cell line source(s)	Expi293F were purchased from ThermoFisher, Catalog Number A14527
Authentication	No further authentication were performed beyond relying on manufacturer CoA.
Mycoplasma contamination	all cell lines tested negative for mycoplasma contamination
Commonly misidentified lines (See ICLAC register)	*Name any commonly misidentified cell lines used in the study and provide a rationale for their use.*

Animals and other organisms

Policy information about studies involving animals; ARRIVE guidelines recommended for reporting animal research

Laboratory animals	Mice; Rag1-/- , C.Cg-Foxp3tm2Tch/J (FOXP3-GFP), DBAxB6 F1 mice 8 weeks females. C57bl/6, male and femals, 8 weeks. Cynomolgus monkey males 2-4 years old. The animals are on a 12/12 day/night cycle. The temperature is 70-72F. Humidity is kept between 30-40%.
Wild animals	The study did not involve wild animals
Field-collected samples	study did not involve.
Ethics oversight	All animal experiments were performed in accordance with federal, state, local and institutional IACUC policies. These policies are determined by the USDA and are further enforced by the MSPCA and Cambridge Commission. Moderna is regularly monitored to ensure compliance to these rules

Note that full information on the approval of the study protocol must also be provided in the manuscript.

Flow Cytometry

Plots

Confirm that:

- [x] The axis labels state the marker and fluorochrome used (e.g. CD4-FITC).
- [x] The axis scales are clearly visible. Include numbers along axes only for bottom left plot of group (a 'group' is an analysis of identical markers).
- [x] All plots are contour plots with outliers or pseudocolor plots.
- [x] A numerical value for number of cells or percentage (with statistics) is provided.

Methodology

Sample preparation	Organs were mechanically disrupted using a sterile syringe plunger through a 70m nylon strainer (Falcon, Catalog #352350) to obtain single cell suspensions. Single cell suspensions were resuspended in 50L of surface antibody staining solution, diluted in PBS + 2% FBS + 1mM EDTA (PBSFE), and incubated for 60 minutes at 4C on a shaking platform. Cells were washed, fixed and permeabilized. Finally, cells were stained with the intracellular target antibodies overnight (12-16 hours) in antibody solution diluted in permeabilization buffer (Biolegend, Catalog #424401). For intracellular cytokine detection, single cell suspensions were resuspended in 100L of RPMI1640 + 5% FBS and PMA/ionomycin activation cocktail (Biolegend, Catalog # 423302) and incubated at 37C, 5% CO2. After 1 hour, 10L of a 10x solution of Brefeldin A (Biolegend, Catalog #420601) and Monensin (Biolegend, Catalog #420701) diluted in RPMI1640 + 5% FBS were added and cells were incubated for an additional 4 hours. Next, cells were stained intracellularly as described above. Samples were acquired on a BD Fortessa flow cytometer. Antibodies were purchased from Biolegend and BD Biosciences and were used at optimized dilutions. Illustrative flow cytometry gating is show in Supplementary Fig. S16. Antibody-fluorophore combinations, clones and dilution are reported in Table S2.
Instrument	Samples were acquired on BD Fortessa and Cytek Aurora.
Software	FlowJo v10
Cell population abundance	For the single cell RNA sequencing experiment, after magnetic bead negative isolation, the population were 98.3% CD4 T cells.
Gating strategy	FSC/SSC gating was used to isolate lymphocytes, then FSC-A/FSC-H for single cell, and then live cells were gated on SSC-A/ APC-Cy7(Viability Dye).

- [x] Tick this box to confirm that a figure exemplifying the gating strategy is provided in the Supplementary Information.
